# Porous Silicon and Silicon Nanowires for On-Chip Supercapacitor Electrodes: A Review

**DOI:** 10.3390/nano15231826

**Published:** 2025-12-02

**Authors:** Daria M. Sedlovets

**Affiliations:** Institute of Microelectronics Technology and High-Purity Materials, Russian Academy of Science (IMT RAS), 6 Academician Ossipyan Str., 142432 Chernogolovka, Moscow, Russia; sedlovets@iptm.ru

**Keywords:** supercapacitors, on-chip devices, porous silicon, silicon nanowires, structure, morphology, coatings, electrodes, electrochemical capacitance, cyclic stability

## Abstract

Finding efficient ways to store energy is a current topic both at the macro level and at the microscale. As silicon plates are the main platform for the integration of microelectronic devices, it is reasonable to use the silicon structures as the active materials for on-chip microcapacitors. Porous silicon (pSi) and silicon nanowires (SiNWs) are ideal candidates for planar electrodes because these layers are directly embedded into the silicon wafer. The review contains a brief summary of the formation features of pSi/SiNW and their electrochemical performance. The structural characteristics of the silicon matrix (depth and morphology) that influence capacitive electrode properties are examined comprehensively for the first time. Particular attention is given to additional coatings on the pore/wire surface. Various ways of depositing metal, carbon, and polymer layers are considered in detail. Different approaches to filling the silicon matrix are explored. Focusing on pSi/SiNWs coatings allows us to identify the effect of the structure, crystallinity, and methods of additional layer deposition on capacitance, cycling stability, and charge transport of modified electrodes. Although fabrication processes for planar microcapacitors based on pSi/SiNWs are currently underdeveloped, the specific requirements and possible challenges of on-chip integration are discussed and proposed.

## 1. Introduction

The investigation of alternative energy sources and the search for efficient ways to store energy are critical challenges. In this regard, electrochemical supercapacitors are of increasing interest to researchers. Here, we focus on micro devices. As silicon wafers are the main platform for integrating microelectronic components, porous silicon (pSi) or silicon nanowires (SiNWs) are promising materials for on-chip microcapacitor electrodes. Recent reviews relate either to various energy devices based on pSi/SiNWs [1,2,3] or to supercapacitor applications using various silicon-based materials [4], so pSi/SiNWs capacitors are considered in less detail than we would like. While the mentioned works certainly cover the main achievements in this field, they do not provide a comprehensive overview. Here, we summarize the data on the electrochemical properties of pSi and SiNWs depending on the structure and morphology of the Si matrix, to identify factors that influence their performance. Particular attention is given to additional coatings on the pore/wire surface because they have a great impact on capacitive properties. Various ways for deposition of metal, carbon, and polymer layers are considered in detail. Key coatings for pSi/SiNW electrodes and their features in the context of microcapacitor performance are summarized. Cyclic stability is especially discussed.

We focus on on-chip applications because in this case, the creation of active material in the body of a silicon wafer is an inspiring idea. Although fabrication methods for planar microcapacitors based on pSi/SiNWs are currently underdeveloped, the specific requirements and possible challenges of on-chip integration are discussed and proposed.

## 2. pSi/SiNW Formation

pSi and SiNWs are structurally similar materials. The main difference between them is their production methods: anodic etching (AE) in hydrofluoric acid solutions/deep reactive ion etching (DRIE) for pSi and metal-assisted chemical etching (MACE)/vapor–liquid–solid (VLS) for SiNWs. We will not focus on these methods—they are discussed in sufficient detail in the relevant reviews: [5,6,7] and [8,9,10], respectively. Here, we briefly describe each approach.

### 2.1. AE

Generally, pSi is formed by anodizing a silicon wafer in an HF-containing electrolyte [5]. Theoretical studies associate the generation of etching sites with the instability of the physical solution–crystal interface under small disturbances in electrolysis processes [11]. The author of [12] argues that, during anodic treatment of the silicon crystal, narrow “channels” form under the influence of HF and move mainly into the depth of the sample. Various models describe the formation mechanism of pSi [13]:

• The Beale model—electric field lines concentrate at surface irregularities, focus current flow at the pore tips, and locally enhance dissolution there.
• Diffusion-limited model—during pore generation, a hole diffuses to the silicon surface and reacts with a Si surface atom. The pore tips are the most likely contact site for particle diffusion.
• Quantum model—the increase in the pSi band gap significantly reduces the concentration of mobile charge carriers up to “depletion”. The current is then limited to the pore tips by increasing the electric field, and the porous structure is passivated by the quantum effect.

The dissolution of silicon during the etching process follows two main paths of conversion of the unstable silicon divalent ion [14,15]:

(1) Si^2+^ + 2H^+^ → Si^4+^ + H_2_,

(2) Si^2+^ + Si^2+^ → Si + Si^4+^.

In the oxidation reaction (1), silicon oxide or a water-soluble H_2_SiF_6_ is generally formed. Disproportionate reaction (2) yields secondary silicon atoms that are resistant to dissolution and unable to integrate into the crystal lattice. These atoms form small nanocrystallites, in which no free holes are available due to the increased band gap [16]. This insulating powdery material is called amorphous silicon or α-Si [17,18].

The morphological characteristics of pSi (the predominant direction of pore growth, their size distribution, and branching) are determined by numerous factors, including silicon wafer characteristics, etching current density, and electrolyte concentration [6].

### 2.2. DRIE

DRIE can also be used to obtain silicon nanorods (SiNRs). DRIE is performed on an inductively coupled plasma system. As described in [19], the plasma is generated through a coil at 13.56 MHz. The platen is powered independently with a second generator at the same frequency.

### 2.3. MACE

The MACE approach for SiNWs is realized by selectively etching silicon in the presence of a catalytic metal.

The two-step MACE method includes systematically depositing a thin layer of metal (usually silver, gold, or platinum) onto the surface of silicon, followed by immersing the sample in an etchant solution containing HF and an oxidizing agent such as H_2_O_2_ [1]. Silicon can be considered as a local anode and the metal as a cathode for the current produced at the Si/metal interface [20]. The anodic reaction proceeds as follows:

(3) H_2_O_2_ + 2H^+^ → 2H_2_O +2h^+^.

As suggested by the authors [21], the cathode reaction occurs as the usual reduction of protons to hydrogen:

(4) 2H^+^ → H_2_↑ + 2h^+^ .

The reduction of the oxidant species generates holes that are injected into the silicon underneath the metal. Hence, in the Si anode region, the silicon is oxidized and dissolved. The dissolution models proceed as reactions I, II, and III, respectively.

Reaction I:

(5) Si + 4h^+^ + 4HF → SiF_4_ + 4H^+^,

(6) SiF_4_ + 2HF → H_2_SiF_6_.

Reaction II:

(7) Si + 4HF_2_^–^ → SiF_6_^2–^+2HF+ H_2_↑ + 2e^–^.

Reaction III:

(8) Si + 2H_2_O → SiO_2_+4H^+^ + 4e^–^,

(9) SiO_2_ + 6HF → H_2_SiF_6_ + 2H_2_O.

In Reaction I, Si is dissolved directly in its tetravalent state without forming silicon dioxide. In Reaction II, the direct dissolution of Si is still present, but in the divalent state. In Reaction III, the Si atoms in contact with the metal are oxidized and then dissolved in two different processes [8].

The single-step MACE concept is based on the use of silver salt. After the cleaning procedure, the sample is immersed in an aqueous AgNO_3_:HF solution. The AgNO_3_ catalyst precipitates, forming Ag nanoparticles (NPs) that are randomly distributed on the silicon flat surface, catalyzing the HF-driven Si etching in a manner similar to that described for H_2_O_2_. Indeed, AgNO_3_ acts as both an oxidant and a metal source [20].

The morphology and characteristics of the resulting SiNWs are influenced by several parameters: the concentration and stirring intensity of the etchant solution, the thickness of the metal catalyst layers, the etching temperature and time, and the doping level of the silicon substrate. The review [1] summarizes the main patterns. The characteristics of SiNWs achieved under various conditions of the MACE process are as follows.

(1) Higher aspect ratio: thicker metal, longer time, increased stirring, higher doping level, and larger etchant concentration;
(2) Larger diameter: thicker metal, higher temperature, increased stirring, and lower doping level;
(3) Higher length: longer time;
(4) Higher etching rate: higher temperature.

The MACE method can be modified into the metal-assisted anodic etching (MAAE) approach. An Au film is deposited on a silicon wafer and patterned using interference lithography to generate an ordered array of holes in the metal. Then, anodic contact is made to the Au mesh, and a Pt wire is used as a counter electrode to complete the circuit. When a bias is applied between the two electrodes in an aqueous HF solution with a concentration of 4 mol/L (M), the etching at the Au–Si interface leads to the formation of SiNW arrays [22].

### 2.4. VLS

VLS has long been the most widely used approach for SiNW fabrication [20]. The growth of nanowires is catalyzed by metal droplets that are realized as a product of the melting of metal NPs contaminated with silicon atoms [9,23]. The most commonly used catalyst remains gold due to the high crystalline quality of the resulting SiNWs and the simple thermodynamics of the Si/Au alloy. The droplets of gold–silicon alloys are liquid at the eutectic phase obtained through specific growth conditions. When the silicon concentration in the alloy exceeds the Si concentration at thermodynamic equilibrium of the eutectic phase, the silicon precipitates under the gold, solidifies, and grows as nanowires [9,23,24].

The standard process for SiNW growth is as follows. A very thin gold film (4 nm [25,26,27,28] or 12 nm [29]) is deposited via evaporation [25,26,27,29] or from gold colloid solution [28,30]. It should be noted that after gold deposition from AuCl_4_ solution [31,32], the grown SiNWs yield a reduced capacitance of several hundred μF/cm^2^. Chemical vapor deposition (CVD) is carried out through exposure to silane gas at low pressure and relatively high temperature (mainly, 500 °C [25,26,28]), except for work [33] where evaporated Sn acts as the catalyst at 1700 °C. To dope the SiNWs, the synthesis temperature was raised to 600 °C, and additional gases were passed (PH_3_ [29,30] and B_2_H_6_ [27] for n- and p-doping, respectively, and H_2_ as the carrier gas in both cases).

### 2.5. Comparison

**Figure 1:**
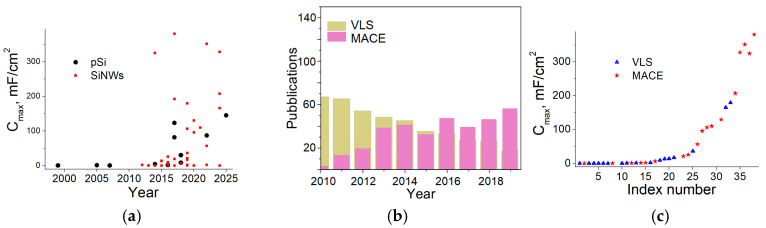
(**a**) Capacitance values of pSi and SiNWs in chronological order. (**b**) Bibliometric analysis of MACE compared to VLS. (**c**) Capacitance values of SiNWs obtained by MACE and VLS in increasing order of magnitude. (**a**,**c**) The data with reference numbers are given in the Supplementary Materials (SM), Section S1. (**b**) Ref. [20] is reproduced with permission, Copyright © 2021 by the authors.

Figure 1a shows that the number of works devoted to SiNW-based supercapacitors exceeds those devoted to pSi-based supercapacitors. It also follows from Figure 1a that the anodic etched pSi electrodes were first proposed in 1991 and have been continuously improved over time. Two notable exceptions to this pattern occurred in 2017 and relate to DRIE-etched pSi [34,35]. SiNWs were first used for microcapacitors in 2012 and generally exhibited capacitive characteristics across a wide range of values.

As can be seen in Figure 1b, there is a change in trend between the MACE and VLS approaches to obtain SiNWs over the past few years. This could be attributed to several drawbacks of VLS, which can be overcome by the MACE process [20]. Indeed, the VLS process, carried out through CVD, has significant disadvantages compared to MACE, as it requires complicated conditions: low pressure, relatively high temperatures, and toxic gases. For these two approaches, an impact on the achieved capacitance is also observed. Figure 1c shows that VLS primarily yields capacitances between 0.013 [36] and 36.3 [26] mF/cm^2^, while MACE primarily enables capacitances of 100 mF/cm^2^ or higher. Therefore, the MACE approach is not only simpler but also more suitable for capacitor applications than the VLS approach (due to the morphology of the resulting SiNWs, as will be discussed in Section 5). It should be noted that the data presented in Figure 1c relate not only to different methods of obtaining SiNWs but also to different measurement conditions, the electrolyte used, additional coatings, and so on. However, the pattern is obvious; works [25,28] are the only exceptions. In these works, high capacitance values were obtained using the VLS approach and by depositing additional layers: amorphous NiB and crystalline CrN, respectively. The influence of the various coatings on pSi/SiNWs capacitances will be studied in more detail in Section 6.

## 3. Electrochemical Performance

There are two main mechanisms for charge accumulation in supercapacitors:

(1) An electrical double layer (EDL) is formed due to electrostatic attraction between the charged electrode surface and the counter ions of the electrolyte. EDL-materials exhibit a close-to-rectangular shape of cyclic voltammograms (CVA) and a linear galvanostatic charge–discharge (GCD) curve;
(2) Pseudocapacitance arises from surface redox reactions, which can cause deviation of the CVA form from a rectangular shape or even the appearance of peaks on the CVA.

While ideal supercapacitors (which operate via the EDL mechanism) can be clearly differentiated from rechargeable batteries (which rely on faradaic processes), pseudocapacitors fall somewhere in between and are not always easy to distinguish [37,38]. The pseudocapacitive behavior is caused by redox reactions, but only on the surface (surface of the pores or SiNWs), in contrast to battery-like materials, where solution-based faradaic processes occur (with redox reactions taking place in the volume). This difference can be determined analytically using the well-known Randles–Sevcik equation: the linear relationship between the peak currents and the scan rate (for the capacitive type) or the square root of the scan rate (for the battery type) [39,40,41].

The specific capacitance is calculated by integrating the area under the CVA curve to obtain the charge value and then dividing this by the surface area, the scan rate, and the potential window. Notably, when evaluating the capacitance, as well as energy and power densities from GCD data, it is necessary to use integral formulas (Equations (10)–(12)) if the GCD curves deviate from the linear pattern [42,43]:

(10) $$C=\frac{2I_{c}\int Vdt}{{A\cdot (∆V)}^{2}},$$

(11) $$E=\frac{I_{c}\int Vdt}{A\cdot 3600},$$

(12) $$P=\frac{I_{c}\int Vdt}{A\cdot ∆t},$$

where C is the area-normalized capacitance in F/cm^2^, Ic is the constant current density in A/cm^2^, ∫Vdt is the integrated area of the discharge curve, ∆V is the scanned potential window in V, A is the area exposed to the electrolyte in cm^2^, E is the energy density in Wh/cm^2^, P is the power density in W/cm^2^, and Δt is the discharge time in s.

**Figure 2:**
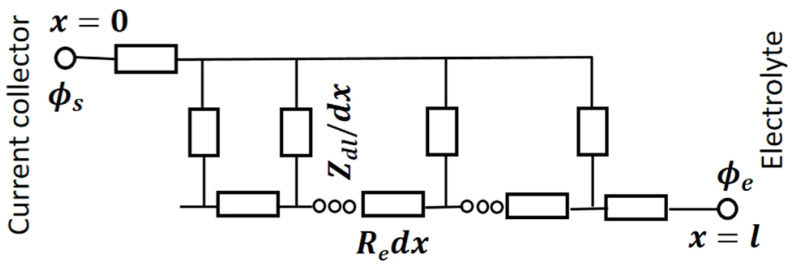
The equivalent electrical circuit of a cylindrical pore with uniformly distributed electrolyte resistance R_e_, and the specific impedance of the interface between the electronic phase and the electrolyte phase, Z_dl_. Ref. [44] is reproduced with permission, Copyright © 2020 by the authors.

**Figure 3:**
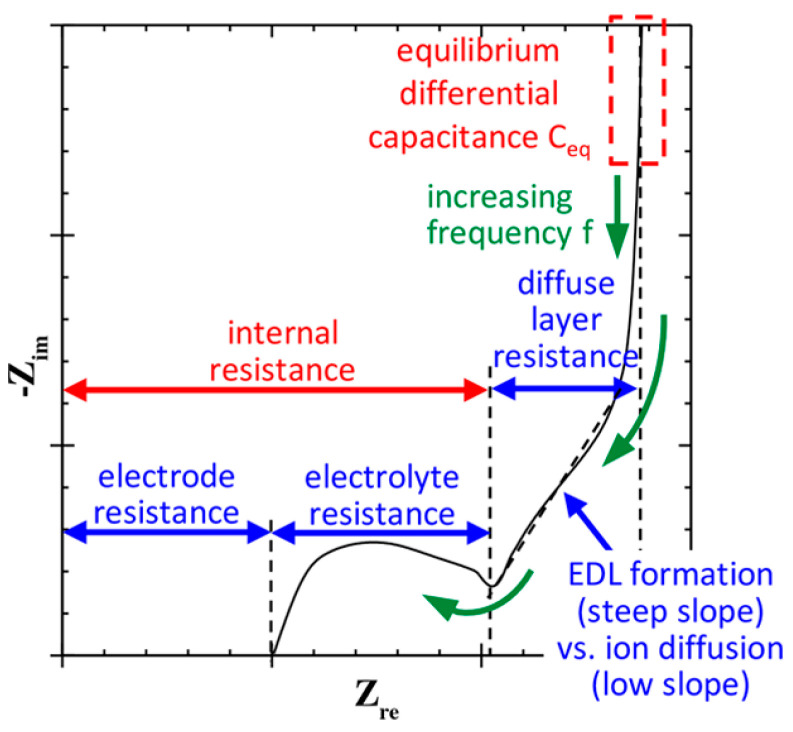
Schematic of typical Nyquist plots for EDL capacitor electrodes or devices. Ref. [45] is reproduced with permission, Copyright © 2018, American Chemical Society.

Impedance measurements are also used for electrochemical characterization of pSi/SiNW structures. Figure 2 depicts the equivalent electrical circuit of the porous electrode (the scheme is described in the Supplementary Materials, Section S2) [44]. Electrochemical impedance spectroscopy (EIS) consists of plotting the so-called Nyquist plots representing the negative of the imaginary part versus the real part of the complex impedance of individual electrodes or the electrochemical cell (see Figure 3). Interpretations of Nyquist plots are usually based on the use of equivalent circuits, but study [45], based on the modified Poisson−Nernst−Planck model, established that the electrode resistance, electrolyte resistance, and the equilibrium differential capacitance can be identified directly from Nyquist plots without relying on equivalent circuits.

The intersections on the *x*-axis at high frequency values are typically called solution resistance (R_s_). The contribution of R_s_ can come from the electrolyte part, the electrode part, and the electrode–electrolyte interface. The semicircle’s diameter shows electrode resistance or R_ct_. In the active compound, R_ct_ takes place in the high-frequency region due to the resistance of charge transfer. Capacitive region or battery-type behavior may be demonstrated by the slope at low frequency [46]. A higher curve slope is usually related to the presence of diffuse hindrances. The Rs and Rct values depend on too many factors and do not always correlate with the capacity value. Therefore, they cannot be used to evaluate charge transfer during the comparisons of samples measured under different conditions (reported in different works). However, comparing different materials (e.g., pSi/SiNWs with various coatings) within one study provides useful information. The frequencies are mainly studied between 1 mHz [47] and 7 MHz [48].

## 4. SiNW Length and PSi Depth

**Figure 4:**
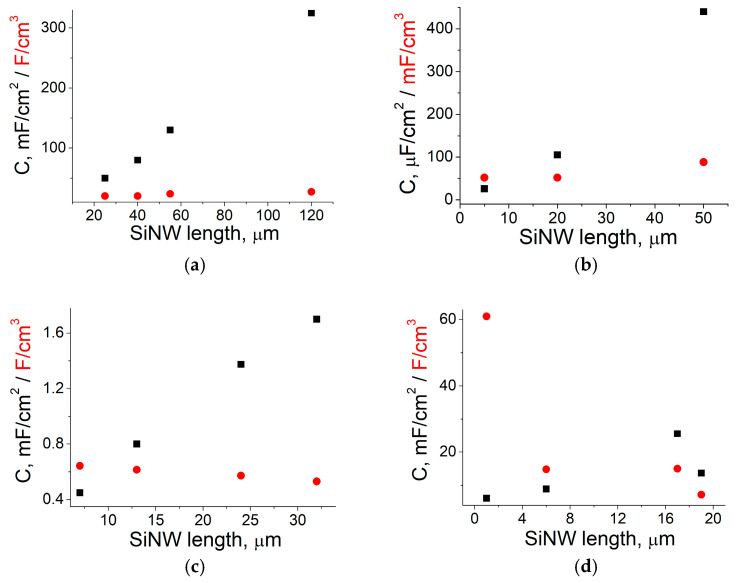
Area-normalized and volumetric capacitances depending on pSi depth/SiNW length. The data from (**a**) ref. [49]; (**b**) ref. [50]; (**c**) ref. [51]; and (**d**) ref. [48].

To determine the influence of SiNW length on their electrochemical performance, we cannot combine the data obtained by different authors because too many factors, in addition to depth, affect the capacitance. Therefore, we consider the capacitance dependence on pSi depth/SiNW length reported in each work. Figure 4 shows some results on area-normalized values of the SiNWs versus the volumetric values, which were calculated by dividing the same magnitude by the SiNW length.

As can be seen in Figure 4a,b, the volumetric capacitance increases together with the area-normalized capacitance. This indicates a qualitative improvement in the material: longer nanowires probably have a larger surface area due to an increased aspect ratio. Otherwise, as shown in Figure 4c, the increase in area-normalized capacitance with increasing SiNW length may be accompanied by a decrease in volumetric capacitance. This pattern means that no qualitative improvement is observed: the area-normalized capacitance growth arises from an increase in the volume of active material, while the area occupied by the electrode on the plate remains the same (i.e., the surface area in contact with the electrolyte). Nevertheless, longer SiNWs are preferable because they exhibit improved capacitance, even if this is only a quantitative effect.

However, the optimal length of SiNWs is often shorter than the maximum (see Figure 4d). Namely, when a critical value is exceeded, the area-normalized capacitance, which has been growing up to that point, begins to decrease. As reported in work [48], increasing the SiNW length above a critical value leads to wire bending, which probably causes a loss of vertical alignment, making it more difficult to form an additional layer on the SiNWs. The longest SiNWs can also be associated with a lengthened pathway for electron diffusion [52]. The longer wires allow for a larger surface area for loading more active materials, but the pathways of electrons are also increased, increasing the electron transport resistance [53]. The inability to fully cover long SiNWs may degrade the electrochemical performance of the composite with polyaniline (PANI) [54]. Similar results are obtained by Maboudian’s group: the maximum capacitance (325 mF/cm^2^) is achieved by coating 120 μm SiNWs with carbon in work [49], but subsequent deposition of an additional layer of MnO_x_ is carried out on the shorter (only 80 μm) C/SiNW structure and yielded 381 mF/cm^2^ [55].

The authors of [56] report increasing capacitance with increasing depth of the porous layer for both pSi and pSi with a graphene-like coating (GLC). The maximum depth of pSi is limited by the geometry and structure of pSi samples when they are exposed to high temperatures and pressure drops during GLC synthesis. Samples with a 150 μm thick porous layer do not change noticeably in geometry or structure; layers with a thickness of 180 μm are characterized by deformation; and cracking of the porous layer occurs when its thickness is increased to 200 μm. Work [56] also shows that the capacitance of intrinsic pSi increases up to great depths (150–180 microns), but the charge accumulation mechanism switches from a capacitive to a battery type, which is also inappropriate. Most likely, this is due to the difficulty of completely impregnating the structure with the electrolyte. Notably, the samples have been exposed to the electrolyte at room temperature for seven or more hours before measurements to ensure satisfactory wettability of the electrolyte to the electrode and, therefore, to obtain accurate results. Boiling in 3 M H_2_SO_4_ solution was also used to enhance impregnation, but the results were practically the same as those obtained through simple exposure at room temperature. As discussed in [57], the porosity of electrode materials leads to surface nonequipotentiality. It can complicate the formation of the EDL and cause nonlinearity in charge characteristics, which is not necessarily associated with Faradaic processes.

## 5. Morphology

**Figure 5:**
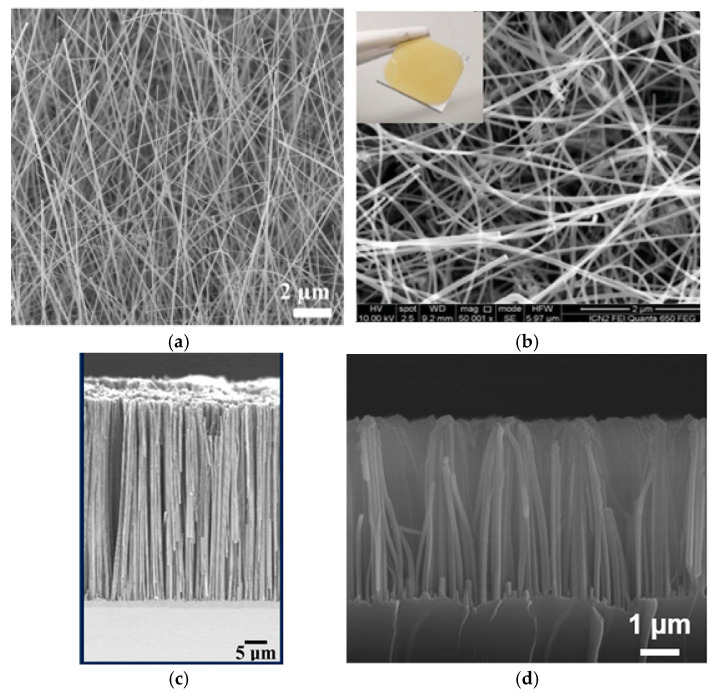
Characteristic scanning electron microscope **(**SEM) images of (**a**,**b**) VLS-grown disorderly interwoven SiNWs and (**c**,**d**) vertically aligned SiNWs obtained by MACE. (**a**) Ref. [58] is reproduced with permission, Copyright © 2016, American Chemical Society; (**b**) ref. [30] is reproduced with permission, Copyright © 2015, The Author(s); (**c**) ref. [49] is reproduced with permission, Copyright © 2014, American Chemical Society; (**d**) ref. [54] is reproduced with permission, Copyright © 2020, American Chemical Society.

As discussed in Section 2.5, MACE provides a higher capacitance for the obtained SiNWs than VLS. Indeed, VLS yields chaotically oriented SiNWs that are either straight (as in Figure 5a) or curved and tangled (as in Figure 5b). In contrast, MACE provides vertically aligned SiNWs in a dense, ordered array (see Figure 5c) or SiNWs stuck together at the top (see Figure 5d).

The superior capacitances (more than 300 mF/cm^2^) achieved by Maboudian’s group [49,55] were also based on ordered oriented thin and dense SiNWs. In contrast, Shen’s group [59,60] used a fundamentally different approach. They obtained short and relatively sparse wires instead of an array. An additional tetramethylammonium hydroxide (TMAH) process was used to reduce the density of the SiNWs. The subsequent goal was not to coat each nanowire; the silicon matrix acted as a scaffold to be filled with active material. This interesting technique yielded a capacitance of more than 300 mF/cm^2^. Section 6.4 will describe Shen’s group’s path in detail.

A similar approach was demonstrated in work [61], where the strategy was also to fill voids inside the nanostructured silicon mesh with an active material. A combination of VLS-grown SiNWs, covered with a protective alumina layer, embedded in a gelatinous-like polymer matrix was successfully achieved. The flexibility of the SiNWs ensures the proper electrical and mechanical connection between the nanowires and the electrochemically active polymer material while allowing a high mass loading of the nanostructure without breaking it. While not very high capacitance values were achieved, the gelatinous-like polymer matrix provided excellent cyclic stability of the composite.

The first papers on pSi microcapacitors reported capacitances of less than 1 mF/cm^2^ for micron-sized pores [62,63]. In recent works, improved capacitances of about 100 mF/cm^2^ and higher have been achieved on DRIE-etched SiNRs (with diameters of several thousand nanometers) [34,35] and then on anodic etched pSi with nm-sized pores [56,64].

**Figure 6:**
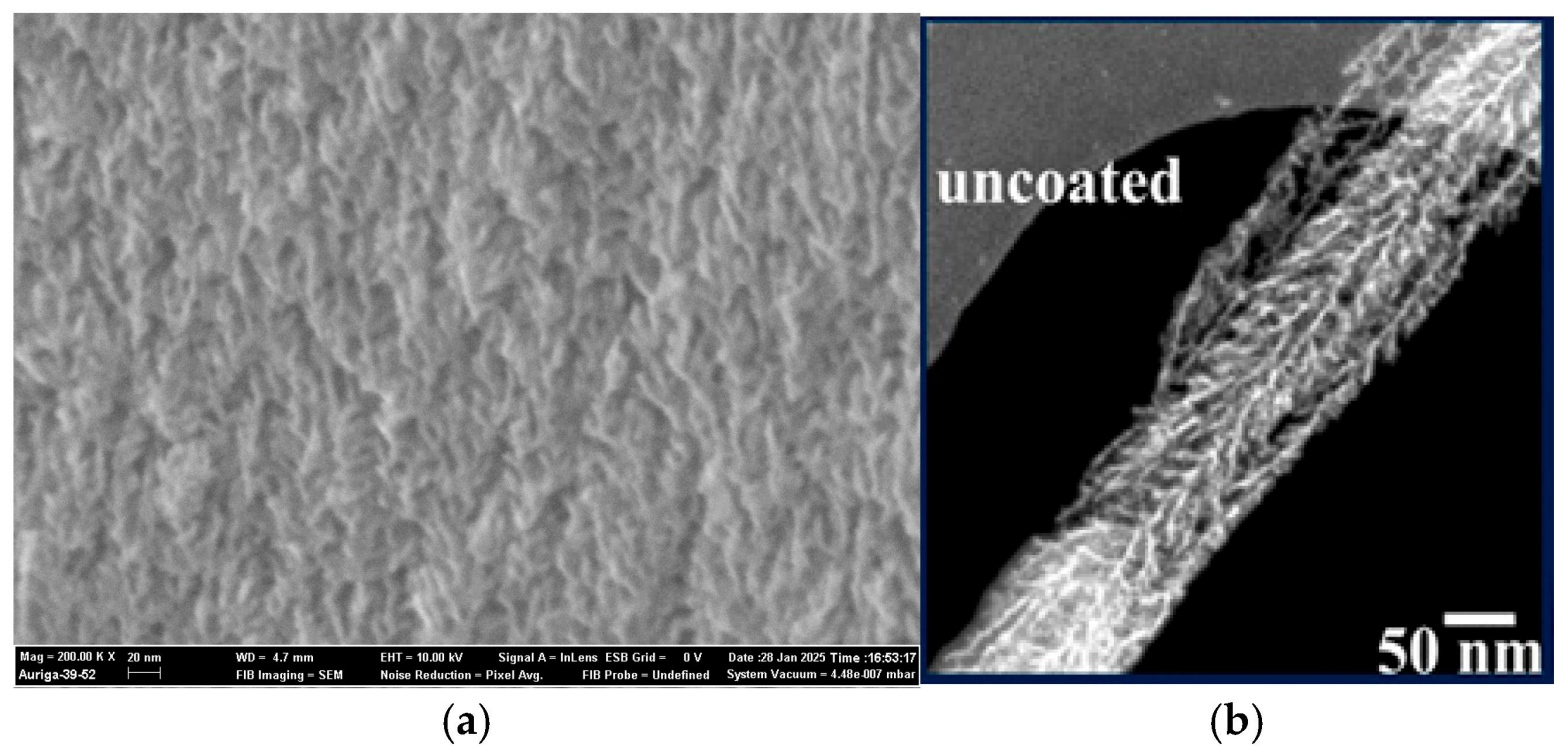
SEM images of (**a**) pSi (145 mF/cm^2^ capacitance with N-doped carbon coating) and (**b**) SiNWs (381 mF/cm^2^ capacitance after coating with carbon and MnO_x_). (**a**) ref. [65] is reproduced with permission, Copyright © 2025 Elsevier Ltd. and Techna Group S.r.l.; (**b**) ref. [49] is reproduced with permission, Copyright © 2014, American Chemical Society.

A distinctive feature of pSi/SiNW nanostructures was found: a herringbone-like morphology is observed in both the most capacitive pores [64] and the most capacitive individual wires (see Figure 6a and Figure 6b, respectively). This demonstrates that the herringbone structure can work successfully at the nanoscale despite the difficulty of electrolyte impregnation if other conditions are properly selected.

The formation of nanocrystallites on the pore surface was observed in work [65]. The disproportionate reaction (2) yielded secondary silicon atoms that are resistant to dissolution and unable to integrate into the crystal lattice. These atoms formed small nanocrystallites (α-Si), which could significantly affect electrochemical performance and were destroyed during pulsed photonic annealing. A similar result was obtained by Wu et al. [66], but they reported pore fusing with increasing annealing temperature. A more detailed study of this phenomenon needs to be conducted, since pSi is a promising material for microcapacitors, although less represented in the literature.

## 6. Coatings

The deposition of various coatings in a complex structure of pSi/SiNWs structures is an interesting challenge. Additional layers have a significant impact on pSi/SiNW capacitive properties. Below is an overview of the coatings, grouped by material type. The following aspects are considered for each coating: deposition method, composition analysis, and influence on pSi/SiNWs’ electrochemical characteristics (maximum capacitance achieved, which is just over 1 mF/cm^2^, and cyclic stability).

### 6.1. Metallic Coatings

Ru:

The ruthenium-coated SiNWs were obtained in work [26] through a simple electroless layer deposition (ELD) process using ruthenium salt. The SiNW substrate was immersed in an aqueous solution of RuCl_3_·H_2_O (1 mM) and HF (0.15 M) for different times at room temperature. The sample was then rinsed with water and dried under a gentle stream of nitrogen. The result was the deposition of the ruthenium NPs consisting of small nanocrystallites on the silicon surface. Elemental mapping and X-ray photoelectron spectroscopy (XPS) ruled out the surface oxidation of the Ru NPs. High-resolution transmission electron microscopy (TEM) and X-ray diffraction (XRD) analyses exhibited clear fringes for Ru NPs, with a lattice spacing of 2.3 Å, which corresponds to the (100) interplanar distance of hexagonal ruthenium. The specific capacitance values increased from 12.5 to 36.25 mF/cm^2^ (at 1 mA/cm^2^) upon increasing the Ru NPs deposition time from 30 to 75 min. An increase in capacitance was explained by the increase in Ru NPs density observed in SEM images. Excellent capacitance (112 mF/cm^2^ at 0.1 mA/cm^2^) and cyclic stability (80% capacitance retention after 50,000 cycles) were achieved.

#### 6.1.1. Transition Metal Oxides

RuO_x_:

Ruthenium oxide was deposited by Zheng et al. [22] on the SiNWs via atomic layer deposition (ALD). In a commercial ALD chamber, diethylruthenocene was heated to 110 °C, and oxygen at room temperature served as the precursor. Different numbers of ALD cycles were used to create layers of varying thickness and continuity. The growth temperature was kept at 290 °C. XPS analysis revealed that the samples contained ~40% oxide phase and ~60% metallic ruthenium. EIS measurements showed that the contribution of Si to the overall electrode performance was negligible for electrodes with 400 ALD cycles. Capacitance was increased by over two orders of magnitude (to 19 mF/cm^2^ with 92% capacitance retention after 10,000 cycles), compared to bare Si electrodes. This increase in capacitance was due to the relatively continuous RuO_x_ coating on the nanowire surfaces, which was uniform from top to bottom and prevented the Si surface from contacting the aqueous electrolyte.

MnO_x_:

The MnO_2_ dispersion was prepared using the electrophoretic deposition (EPD) method in work [33]. Manganese sulfate (MnSO_4_), ammonium persulfate ((NH_4_)_2_S_2_O_8_), and ammonium hydroxide (NH_4_OH) were used as the manganese source, oxidizing agent, and precipitating agent, respectively. Then, a manganese oxide shell layer was deposited on SiNWs from the obtained solution by an EPD process at room temperature. XRD confirmed the presence of the MnO_2_ NP coating on the SiNWs. Excess mass loading (30 s of EPD) decreased the specific capacitance of the electrode. The components in the core–shell structure greatly affected performance: the MnO_2_ NPs (deposited for 15 s) stored charge, and the SiNWs offered a shorter pathway for charge transport to the electrode. However, the overall capacitance (2.1 mF/cm^2^) of the MnO_2_ NPs-modified electrodes was not particularly high.

Dubal et al. [30] grew ultrathin MnO_2_ nanoflakes on SiNWs using a simple ELD method. They mixed a 50 mL solution of 2 mM potassium permanganate (KMnO_4_) with 2 mL of 98 wt% hydrochloric acid (HCl). A silicon wafer with pre-deposited SiNWs was then immersed in the bath at 323 K for 5, 10, 15, or 20 min. Finally, the MnO_2_/SiNW substrates were removed, rinsed, and dried in a vacuum at 373 K for two hours. The proposed growth mechanism for MnO_2_/SiNWs was as follows: initially, MnO_4_^−^ nuclei were produced, adsorbed on the surfaces of SiNWs, and formed MnO_2_ nuclei upon reduction, which grew, aggregated, and transformed to nanoflakes. Ultimately, the MnO_2_ nanoflakes became compact and completely covered the surface of the SiNWs. XPS investigations indicated that the oxidation state of Mn in the heterostructure was Mn (IV). Selected area electron diffraction (SAED) and XRD data revealed the formation of polycrystalline birnessite-type MnO_2_. The unique 3D mesoporous structure of MnO_2_ on SiNWs provided large-area contact for the electrode and electrolyte; this structure enabled the accommodation of large volume changes and the release of associated strain generated during rapid charge and discharge cycling; the MnO_2_/SiNWs core–shell heterostructure was strongly supported on a silicon wafer, eliminating the need for a polymer binder or conductive additives. The design of the hierarchical MnO_2_/SiNWs core–shell heterostructure was improved by coupling it with a novel, Li-ion-doped ionic liquid electrolyte based on LiClO_4_ and 1-methyl-1-propylpyrrolidinium bis(trifluoromethylsulfonyl)imide (PMPyrrBTA). LiClO_4_, the primary ionic working species, was reversibly inserted into and out of the lattice tunnels between the MnO_6_ octahedral subunits. This caused a large amount of the manganese oxide to participate in surface redox reactions. The capacitance of 13 mF/cm^2^ was achieved with 91% retention after 9000 cycles.

In work [67], MnO_2_ was deposited on SiNWs by ELD from KMnO_4_. Prior to deposition, the SiNWs substrate was immersed in HF (5%) solution to remove the native silicon oxide layer and generate a hydrogen termination (H-SiNWs). The latter plays a key role in the deposition mechanism according to the following reactions [68]:

(13) SiNWs/Si + HF → H-SiNWs/Si

(14) H-SiNWs/Si + KMnO_4_ → MnO_2_/SiNWs/Si

Energy-dispersive X-ray (EDX) analysis confirmed the presence of MnO_2_, while the secondary-ion mass spectrometry depth profiles of Mn, O, and Si atoms revealed that SiNWs were partially oxidized and that MnO_2_ deposition on SiNWs could reach a depth of about 3 μm. The deposition of MnO_2_ on H-SiNWs increased the capacitance to 21.3 mF/cm^2^ [67] due to its pseudo-capacitive effect, wherein the quasi-reversible redox reaction involves the insertion/deinsertion of alkali ions (Na^+^) or protons (H^+^) in the empty sites of the MnO_2_ [69,70,71]:

(15) MnO_2_ + H^+^/Na^+^ + e^−^ ↔ MnOOH/MnOONa

The MnO_2_ deposition process may also involve carbon as a reducing agent [72]:

(16) 4KMnO_4_ + 3C + H_2_O → 4MnO_2_ + K_2_CO_3_ + 2KHCO_3_

The redox reaction (16) was carried out by the authors of [55] for MnO_x_ layer deposition on the C/SiNWs by means of an ELD method from KMnO_4_. KMnO_4_ concentrations were varied between 20 to 100 mM, and ELD time intervals were varied between 5 and 60 min. The electron transfer from nanocarbon to MnO_4_^−^ initiated the heterogeneous nucleation of MnO_x_ on the active surface. The formation of MnO_x_ was thus closely related to the carbon structure. XPS studies revealed a predominance of MnO over the Mn_2_O_3_ phase in the MnO_x_ coating layer. The electrochemical analyses indicated that the best performance was obtained on SiNWs with a length of 75 μm and in an 80 mM KMnO_4_ solution for 45 min. The capacitance of 381 mF/cm^2^ was achieved with 84% retention after 5000 cycles. The decrease in capacitance after extended cycling might be due to electrochemical dissolution of the MnO_x_ layer, as described in previous studies [73].

Electrochemical deposition (ECD) and ELD methods were compared in work [34] as routes for manganese oxide deposition onto DRIE-etched silicon taper nanorods (SiTNRs) coated with TiN. The ECD process was realized by a galvanostatic process at a current density of 0.4 mA/cm^2^ for 16 min in an aqueous solution of 0.01 M Mn(CH_3_COO)_2_ and 0.02 M Na_2_SO_4_ at room temperature. ELD of MnO_2_ was performed for 8 h in a 200 mL aqueous solution containing 2 mM KMnO_4_ and 5 mM KOH. Volatile N_2_H_4_ was introduced into the KMnO_4_ solution as the reductant, thus supporting the growth of MnO_2_. EDX analysis confirmed that MnO_2_ was deposited on the SiTNR/TiN structures by using both methods. Both electrodes retained approximately 95.7% of their initial capacitance after up to 5000 cycles, while the main capacitance fading occurred during the first 500 cycles. However, the capacitance value increased noticeably from ~40 mF/cm^2^ (for ECD-deposited MnO_2_) to ~80 mF/cm^2^ (for ELD-deposited MnO_2_) at 10 mV/s.

Section 6.4 will discuss another study in which manganese oxide was combined with other coatings and used as a filler for a sparse SiNW scaffold [59,60,74].

NiO:

N-doped carbon (N-carbon) underlayer was used to prepare SiNWs/N-carbon/NiO electrode [75] through the pyrolysis of polydopamine (PDOP). The SiNWs/PDOP composites were prepared by a modified oxidative polymerization (OxP) method in a quartz glass reactor equipped with a water jacket connected to cooling circulating water pump equipment, which ensured that the polymerization occurred at 0 °C. 100 mg of dopamine hydrochloride, 100 mg of ammonium persulfate, and 60 mL of the phosphate buffer solution (pH = 6.5) were added to the reactor. The reaction lasted for 24 h and was repeated twice. Raman analysis revealed that the carbon coating was predominantly composed of sp^2^-type carbon, and XPS results demonstrated that the calcination treatment had transformed PDOP to the N-carbon. Subsequently, NiO deposition was carried out by ELD method for 10 min in the mixed solution of 20 mL of 1 M nickel sulfate, 16 mL of potassium persulfate, and 4 mL of aqueous ammonia, followed by a one-step calcination treatment in an argon atmosphere at 500 °C for 4 h. Field emission SEM confirmed that the NiO coating consisted of nanoflakes (with a thickness of about 100 nm as measured using TEM). The as-prepared SiNWs/N-carbon/NiO composite electrode showed a specific capacitance of up to 110 mF/cm^2^ with 80.8% retention after 4000 cycles. The N-carbon and nickel oxide played a synergistic role in improving the electrochemical performance in aqueous electrolytes. Enhanced charge transfer arose from the N-carbon coating, while additional pseudocapacitance arose from the highly reversible redox reaction (17): oxidation (forward reaction) and reduction (back reaction) [76].

(17) NiO + OH^–^ ↔ NiOOH + e^–^.

#### 6.1.2. Other Metal Compounds

TiN:

ALD-deposited TiN coating greatly improved the electrode performance by increasing the conductivity and passivating the silicon surface, as has been found by several groups [34,77,78,79]. TiN with near-ideal conformality was deposited via ALD at 450 °C using TiCl_4_ and NH_3_ as the precursors and N_2_ as the carrier gas.

In work [77,78], approximately 6 μm thick pSi was prepared during 30 min anodization at a current density of 5.3 mA/cm^2^ in 50% HF and ethanol solution (1:4). Using two pPSi/TiN electrodes with a polydimethylsiloxane frame as a separator and as a reservoir for electrolyte, the volume-normalized capacitance of 7.3 F/cm^3^ was measured in work [77], and during the 5500 cycles, the capacitance changed very little. We obtained the area-normalized capacitance value by dividing the volume-normalized one by the porous layer thickness (6 μm):

(18) 7.3 F/cm^3^ × (6 × 10^−4^ cm) = 4.38 × 10^−3^ F/cm^2^ = 4.38 mF/cm^2^,

The in-chip electrochemical cell, consisting of two pPSi/TiN electrodes inside silicon plates with an electrolyte reservoir between them, was implemented in work [78]. This device configuration (for more details, see Section 7) allowed the achievement of higher volumetric capacitance (with 70–75% retention after 13,000 cycles) as well as higher energy and power densities.

In the same ALD process, TiN was deposited onto SiNRs fabricated through a cyclic DRIE route (75 etching cycles were performed to obtain SiNRs with a depth as high as ~20 μm) [79]. SEM and EDX studies illustrated the successful growth of TiN on SiNRs. As a result, the capacitance of 1 mF/cm^2^ was achieved with 95.2% retention after 2000 cycles.

The SiTNR/TiN was coated with MnO_2_ through ELD, as described in Section 6.1.1 [34], yielding a capacitance of 81.6 mF/cm^2^ with 95.7% retention after 5000 cycles.

NiB:

The deposition of nickel boride (NiB) onto SiNWs was carried out through ELD [25]. Nickel chloride (NiCl_2_) aqueous solution was mixed with ethylene diamine C_2_H_4_(NH_2_)_2_ and then with NaOH solution containing NaBH_4_ as a reducing agent. The final mixture was heated up to 90 °C, and SiNW/Si substrates were immersed in it for 10, 20, and 30 s. Energy dispersive spectroscopy and XPS data confirmed the NiB formation over the surfaces of SiNWs. SEM results showed uniform coverage of the underlying template along the entire SiNWs length. XRD indicated that the as-deposited NiB is amorphous (the capacitance of crystalline NiB needs to be explored in the future). The reported preparation method led to the formation of an amorphous phase and allowed the achievement of 165.7 mF/cm^2^ capacitance with 93% retention after 15,000 cycles.

CrN:

Chromium nitride (CrN) layers were deposited onto the SiNWs array by bipolar magnetron sputtering [28]. The usage of bipolar sputtering with two chromium targets, instead of conventional sputtering, was beneficial for well-crystallized nanostructures. SEM, electron diffraction, and XRD analyses revealed that the SiNWs were covered uniformly with small cubic CrN crystallites. EIS measurements indicated that the CrN layers reduced the charge transfer resistance and the electrodes became more conductive as the thickness of the CrN layer increased from 290 to 900 nm. However, the areal capacitance increased with the CrN thickness up to 550 nm, then stabilized at 180 mF/cm^2^ as the thickness reached 550 nm (the electrode could sustain 92% of its initial capacitance value after 15,000 cycles). A further increase in CrN thickness (from 550 to 900 nm) led to a capacitance decrease, because part of the surface became inaccessible. TEM images showed a herringbone structure due to the deposition of 550 nm CrN on SiNWs. This peculiar nanostructure, which contained highly nanoporous channels, was expected to contribute to the electrode capacitance enhancement. We should recall that the herringbone-like morphology was observed in both the most capacitive AE-etched pSi [64] and the most capacitive MACE-etched SiNWs [55].

### 6.2. Carbon Coatings

Due to its biocompatibility, chemical stability, high specific surface area, and electrical conductivity, carbon is a promising material for improving the properties of pSi/SiNWs for potential applications in optoelectronics [80], sensors [81,82,83,84,85], and photodetectors [86]. Below, various carbon coatings proposed for improving the electrodes of pSi/SiNW microcapacitors are listed and described.

#### 6.2.1. Diamond

Boron-doped diamond coating [87] was synthesized onto SiNWs for 10 min in a microwave (MW) CVD system from a H_2_/CH_4_ gas mixture containing trimethylborane at an MW power of 2750 W, a gas pressure of 40 mbar, and a substrate temperature of 675 ± 10 °C. SEM images showed that a uniform, adherent, and homogeneous coating was deposited along all SiNWs with a thickness of ~100 nm. The nanocrystalline grain sizes varied from tens of nanometers to approximately 150 nm. The boron-based doping and structure of crystalline diamond were confirmed by Raman spectroscopy. A capacitance of 1.5 mF/cm^2^ was achieved with 65% retention after ultra-long cycling measurements (1,000,000 cycles).

In work [88], SiNWs were successfully coated with diamond-like carbon (DLC) films using EPD in a two-electrode electrolytic cell (graphite sheet was used as the anode, SiNWs as the cathode, and dimethylsulfoxide solution as the electrolyte) at 70 °C for 60 min at an applied voltage of 150 V. SEM analysis showed that the DLC films were uniformly deposited on the whole SiNW surface and composed of compact and small spherical grains with uniform distribution. Raman spectroscopy, EDX, and XRD studies indicated that the coating films were DLC. The electrochemical behavior of the SiNWs was clearly enhanced by the DLC coating, showing a specific capacitance of 2 mF/cm^2^ (at 0.01 mA/cm^2^) with capacitance retention of 90% after 16,000 cycles. Notably, a specific capacitance of 400 mF/cm^2^ was achieved at a scan rate of 5 mV/s, but an unusually large difference between CVA and galvanostatic charge–discharge GCD measurements was not explained in the manuscript, so we present the results obtained from GCD here.

#### 6.2.2. SiC

In work [51], a thin (several tens of nm) SiC layer was chosen for chemically passivating the SiNWs using low-pressure CVD from 1,3-disilabutane (5 sccm) at 800 °C and 5 × 10^−6^ Torr for 3 min. A capacitance of 1.7 mF/cm^2^ was achieved for the longest (32 μm) SiNWs with SiC coating, and remained stable (95% retention) over 1000 charge/discharge cycles.

#### 6.2.3. Nanocarbon

A highly activated carbon layer was developed on vertically aligned SiNWs through pyrolysis of glucose followed by annealing at 600 °C [48]. SEM and TEM results revealed that the nanocarbon layer (thickness of ~20 nm) was uniformly coated over the SiNW length (up to 19 μm). The carbon-coated 17 μm thick SiNWs displayed a specific capacitance as high as 25.6 mF/cm^2^ with 75% retention after 25,000 cycles.

Depositing an ultrathin (sub-nm) carbon sheath over the SiNWs enabled full electrolyte access to the porous surface area while still mitigating Si degradation [49]. Carbonization was performed in an atmospheric pressure CVD chamber at 900 °C in a CH_4_/Ar flow for 30 min. Raman spectroscopy data indicated a predominantly sp^2^ hybridized carbon film with a correspondingly high electronic conductivity. The C/SiNW composite yielded 325 mF/cm^2^ with 83% retention after 5000 cycles.

#### 6.2.4. 1D Nanocarbon

In work [35], fullerene-like carbon (FC) decorated carbon nanotubes (CNTs) were used to fill a DRIE-etched SiTNR scaffold. Ni catalysts for CNT growth were deposited by electron-beam deposition at a high vacuum (5 × 10^−6^ Torr). The growth of FC-CNTs was carried out via an atmospheric pressure CVD process in Ar/H_2_/C_2_H_2_ (100:20:1 *v*:*v*:*v*) atmosphere at 750 °C for 12 min. SEM images revealed that the interconnection assembly of CNTs readily created abundant interspaced macropores, while the FC NPs contributed to forming mesopores (2 to 10 nm), as shown by TEM. Altogether, 1D nanocarbons incorporated in a tapered silicon nano-scaffold improved the capacitance (123 mF/cm^2^ with excellent retention of 102% after 5000 cycles).

The CNTs were also used for pSi decoration: new functional nanocomposite materials based on layers of pSi, CNTs, and tin oxide were proposed for supercapacitor electrodes [89]. The SnOx/CNT/pSi hybrid electrode exhibited a specific capacitance of 347 mF/cm^2^, but its cyclic stability was insufficient (50 charge–discharge cycles were carried out to assess the cyclic stability of the measured electrodes).

#### 6.2.5. Graphene-Based Films

The graphenic layer was grown directly on the pSi structure through Ni-assisted CVD at heating temperatures of 1000–1100 °C under an increased pressure of 60 Torr in a CH_4_/Ar flow [66]. The coating consisted of few-layer graphene (FLG), which was confirmed by Raman spectra. High-magnified SEM images showed that after CVD at 1000 °C, the average pore diameter was ~11 nm (in the range of mesopores). An increase in the annealing temperature to 1100 °C caused the pore coalescence, with all mesopores merging to form larger macropores. At the annealing temperature of 1050 °C, some mesopores (but not all) fused together, forming a porous structure composed of both mesopores and macropores. This hybrid-porous FLG/SiNWs enabled achieving a high areal capacitance of 6.21 mF/cm^2^ and an unusual progressive cyclic stability of 131% at 10,000 cycles.

Electropolymerization (ElP) of 2,6-dihydroxynaphthalene (DHN) using CVA or potentiometry with subsequent thermal pyrolysis (800 °C, N2, 4 h) resulted in a GLC strongly adhering to the nanoporous silicon matrix [90]. ElP was carried out using a 2 mM solution of DHN dissolved in the electrolyte solution (10 mM phosphate buffer saline solution, pH 7.40, containing 0.1 M KCl):

- by potentiometry—at 1 mA for 120 s (“J” sample, active mass = 90.8 μg);
- by CVA—at 5 mV/s for 6 cycles (“CV” sample, active mass = 74.2 μg).

After annealing, the capacitances were measured: 162 F/g and 100 F/g for the “J” and “CV” electrodes, respectively. Since the active area was equal to 0.49 cm^2^, the area-normalized capacitances were calculated as follows:

(19) 162 F/g = 162 × (90.8 × 10^−6^ g) × (1/0.49 cm^2^) = 30 mF/cm^2^,

(20) 100 F/g = 100 × (74.2 × 10^−6^ g) × (1/0.49 cm^2^) = 15 mF/cm^2^.

The capacitance retentions after 1000 cycles were equal to 75% and 80% for the “J” and “CV” electrodes, respectively. XRD data confirmed a low graphitization degree of GLC, which may be the reason for poor cyclic stability.

Continuous GLC, consisting of sub-ten-nanometer graphene flakes, was deposited in the pSi structure in a CVD reactor through high-temperature (950 °C), low-pressure (∼100 Pa) pyrolysis of C_2_H_5_OH vapor [56]. For GLC deposition inside the porous structure, a sharp pressure drop (SPD) was required during the synthesis. The SPD mode of CVD allowed the synthesis of GLC throughout the full depth (up to 200 μm) of a complex, herringbone structure of nanopores, which was confirmed by Raman spectroscopy. As a result, the composite electrode showed a capacitance of 87 mF/cm^2^ and retained 100% of its value after 15,000 cycles.

In work [64], the above CVD procedure was carried out (but acetonitrile was used as the precursor) for N-doped GLC (N-GLC) deposition in pSi structures. XPS analysis revealed that the main configuration of the nitrogen atoms embedded in GLC was graphitic. The capacitance increased (up to 145 mF/cm^2^) when pSi was coated with N-GLC due to the enhanced conductivity (namely, improved electron transfer) induced by the graphitic configuration of the N-dopant. Good cyclic stability was also achieved (over 20,000 cycles even at low scan rates).

Carbon undercoating was also used for NiO [75] and MnO_x_ [55] deposition as described in Section 6.1.2, and for PEDOT [58] and PANI [91] deposition, as will be discussed in Section 6.3.

### 6.3. Polymer Coatings

#### 6.3.1. PPy

Polypyrrole (PPy) films were electrochemically deposited onto SiNTrs from a PYR_13_TFSI (N-propyl-N-methylpyrrolidinium bis(trifluoromethanesulfonyl)imide) solution containing 0.1 M Py as the monomer [27]. The ElP was carried out in a three-electrode electrochemical cell using a potentiostat–galvanostat at a constant potential of 0.4 V (vs. Ag/Ag^+^) under a polymerization charge of 750 mC/cm^2^ controlled by the chronocoulometry technique in an argon-filled glove box with oxygen and water levels below 1 ppm. A uniform and homogeneous PPy coating had a globular morphology as illustrated by SEM. The thickness of the conducting polymer coating was determined to be around 20 nm using TEM. From CVA, a pair of redox peaks were detected, which are associated with the oxidation (forward reaction (21)) and reduction (back reaction (21)) of Ppy (intercalation and deintercalation of electrolyte anions, respectively):

(21) PPy + n TFSI^−^ ↔ (PPy)_n_ + (TFSI^−^)_n_ + ne^−^.

The PPy coating increased the capacitance (to 14 mF/cm^2^). However, 30% capacitance loss was observed after 10,000 cycles, whereas bare SiNTr microcapacitors exhibited no loss of electrochemical stability after 10,000 galvanostatic charge–discharge cycles.

#### 6.3.2. PEDOT

The surface functionalization of SiNWs by electroactive conducting polymers was proposed in work [29]. Poly(3,4-ethylenedioxythiophene) (PEDOT) coatings were electrochemically deposited from a propylene carbonate solution containing 10 mM EDOT as the monomer and 0.1 M tetrabutylammonium tetrafluoroborate (TBABF_4_) as the electrolyte at a constant potential of 0.9 V (vs Ag/Ag^+^) for 1 h. SEM study showed that a nanometric PEDOT coating (thickness of 100 nm) with a globular morphology was deposited along all SiNWs, increasing the capacitance to 17 mF/cm^2^.

**Figure 7:**
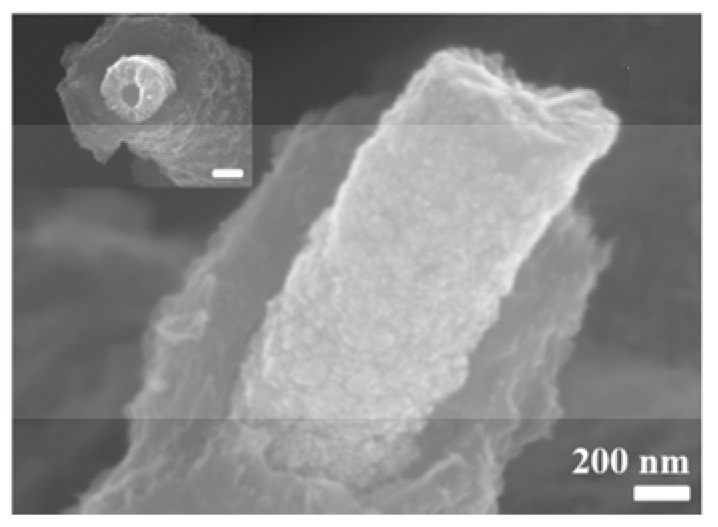
Cross-sectional view of PEDOT/diamond/SiNWs. Scale bar of inset: 100 nm. Ref. [58] is reproduced with permission, Copyright © 2016, American Chemical Society.

In work [58], SiNWs were introduced into an MWCVD system and exposed to a H_2_/CH_4_ gas mixture at an MW power of 2750 W and a temperature of 675 °C to coat the electrode with diamond. Subsequent electrochemical deposition of nanometric PEDOT films was carried out from a PYR_13_TFSI solution containing 0.1 M EDOT as the monomer (as described in [92]). The deposition of diamond and PEDOT was confirmed by Raman spectroscopy and XPS, respectively. Scanning TEM and EDX analyses showed that the PEDOT and diamond conformal coatings were deposited on SiNWs with a 3D multi-hierarchical structure (see Figure 7), which yielded a capacitance of 8.5 mF/cm^2^ (at 1 mA/cm^2^) with 80% retention after 15,000 cycles in butyltrimethylammonium bis(trifluoromethylsulfonyl)imide (N_1114_TFSI) electrolyte.

#### 6.3.3. Combined Polymers

**Figure 8:**
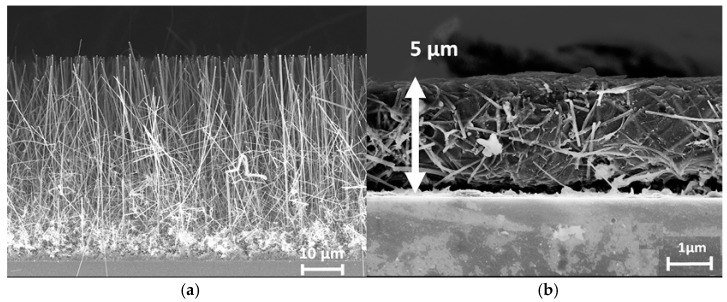
SEM cross-section images of (**a**) pristine Al_2_O_3_/SiNWs and (**b**) gelatinous-like PEDOT-PSS/Al_2_O_3_/SiNW composite. (**b**) Ref. [61] is reproduced with permission, Copyright © 2019, American Chemical Society.

One-step deposition of PEDOT with poly(styrenesulfonate) (PSS) onto Al_2_O_3_-protected SiNWs was reported in work [61]. A 3 nm thick Al_2_O_3_ layer was deposited using ALD from trimethylaluminum and H_2_O at 250 °C under 10^−2^ Torr. By replacing the uncontrolled, native, and inherent Si oxide layer with alumina, the silicon nanostructure was protected from any oxidative stress from contact with the aqueous electrolyte. The conducting polymer layer was deposited by a simple drop of ethanol, followed by three droplets of the homogenized PEDOT-PSS solution. SEM images showed a mesh of highly doped 50 μm long Al_2_O_3_-coated SiNWs (Figure 8a) embedded inside a 5 μm thick gelatinous-like polymer matrix (Figure 8b), ensuring an excellent electrical connection between these two materials. The coating of the polymer flattened the nanostructure, and the flexible nanowires interlocked with each other. This new nanocomposite material exhibited a capacitance of 8.5 mF/cm^2^. Stability was confirmed by a performance decline of only 5% after at least 500,000 cycles. Throughout the cycling, the electrolyte was percolating inside the composite, reorganizing the polymeric matrix by the successive ionic intercalations and deinsertions. The areal capacitance, therefore, increased until the whole nanocomposite was soaked with the electrolyte, where it reached a stable plateau.

SiNWs/PEDOT/PPy composites were obtained consecutively by a photo- and chemical-OxP method [52]. An in situ photo polymerization (PhP) onto hydrogen-terminated SiNWs (H-SiNWs) was carried out via the reaction with a monomer solution (0.005 mol of EDOT, 25 mL of deionized water, and 25 mL of acetonitrile) under UV irradiation at 365 nm for 3 h in a quartz glass reactor equipped with a blue light filter.

After that, the SiNWs/PEDOT electrodes were suspended in 40 mL of monomer solution (1 M HCl containing 0.01% (*v*/*v*) pyrrole), and 10 mL of 1 M FeCl_3_ aqueous solution was added dropwise. The polymerization reaction was carried out at about 0 °C for 12 h. The Raman spectra proved the successful synthesis of PEDOT and PPy in the composites. As indicated by SEM, the SiNWs/PEDOT/PPy had a core–shell structure, and the thickness of the PPy nanolayers was about 40 nm. XPS data confirmed the interaction between PEDOT NPs and SiNWs in the composite (the photoexcited SiNWs directly initiated the PhP of EDOT at the surface of SiNWs, which built up this interaction). The PEDOT NPs with excellent electrical conductivity not only acted as efficient conducting highways for facilitating electron transfer, but also made the surface of SiNWs more compatible with the PPy nanolayers. The composite exhibited a specific capacitance of 106 mF/cm^2^ with a retention of 80.2% after 5000 cycles, and its core–shell structure remained stable after the cycling test.

#### 6.3.4. PANI

The in situ polymerization of aniline over SiNWs was carried out via the OxP process [54]. The SiNW substrate was soaked in 0.5 M H_2_SO_4_ containing aniline as the monomer for 30 min. Consequently, the mixture of 0.03 M ammonium persulfate ((NH_4_)_2_S_2_O_8_) and 0.5 M H_2_SO_4_ was gradually added to the initial solution under an ice-water bath for 4 h. PANI layers of multifarious morphologies covered over SiNW arrays were obtained by varying the aniline concentrations from 0.4 mM to 6.4 mM. Field emission SEM revealed that the coral-shaped PANI/SiNW hybrid material was obtained at the aniline concentration of 1.6 mM. XPS and Raman peaks registered from the composite surface were assigned to the PANI macromolecular chain. Water contact angle measurements demonstrated that modification of SiNWs with PANI led to greater hydrophilicity and therefore accelerated the exchange rate of H^+^ ions during the charge–discharge process. As a result, a capacitance of 81 mF/cm^2^ was achieved with 71.8% retention after 2000 cycles.

The influence of PANI and graphene nanowalls (GNWs) (both separately and together) on the SiNWs’ electrochemical performance was investigated in work [91]. PANI was electrochemically deposited using a monomer solution (a mixture of 0.1 M aniline, 30 mL of ethanol, and 70 mL of 1 M H_2_SO_4_) at a constant potential of 0.8 V (vs Ag/Ag^+^) for 15 min. GNWs were grown on the plasma-etched SiNWs’ surface by an MW plasma-enhanced CVD method from H_2_/CH_4_ for 20 min at an RF power of 700 W and at a chamber pressure of 4 kPa. The capacitance achieved for SiNWs/GNW and SiNWs/PANI was 4.6 mF/cm^2^ and 20.9 mF/cm^2^, respectively. The sequential deposition of both coatings yielded an excellent improvement: the SiNW/GNW/PANI capacitance was 130 mF/cm^2^ with 80% retention after 2000 cycles.

### 6.4. Combined Coatings

The above-discussed combined coatings are mainly carbon layers under transition metal oxides and conductive polymers. The latter are often used in combination with each other.

Here, we describe the original Shen’s group concept that opens up an avenue to prepare 3D silicon nanostructures based on metal oxide and conductive polymers, which not only reduce the contact resistance between the active materials and the current collector but also shorten the ion and electron diffusion paths. Unlike traditional attempts to obtain a dense array of SiNWs and further coat each nanowire, the reduction of SiNW density is achieved in a mixed TMAH solution (TMAH:isopropyl alcohol:distilled water = 0.3:5:25 in volume ratio). It is known that TMAH is an attractive etchant solution for the silicon substrate due to its nontoxic, easy disposal, and high-quality anisotropic features without any metal ion contaminants. The reaction equation between silicon and TMAH is as follows [93]:

(22) Si + 2(CH_3_)_4_NOH + 2H_2_O → 2(CH_3_)_4_N^+^ + SiO_2_(OH)_2_^2−^ + 2H_2_↑.

**Figure 9:**
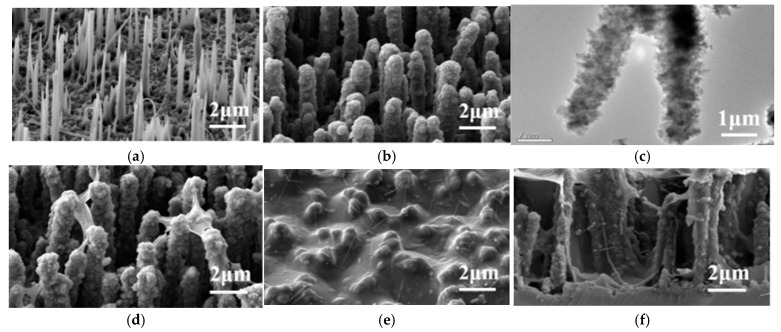
SEM images of (**a**) SiNWs and (**b**) SiNWs/PEDOT + MnO_2_. (**c**) TEM image of SiNWs/PEDOT + MnO_2_. SEM images of (**d**) SiNWs/PEDOT + MnO_2_/rGO and (**e**) SiNWs/PEDOT + MnO_2_/rGO/AgNWs:PEDOT-PSS. (**f**) Cross-sectional SEM image of SiNWs/PEDOT + MnO_2_/rGO/AgNWs:PEDOT-PSS. Ref. [53] is reproduced with permission, Copyright © 2022, American Chemical Society.

In work [53], the silicon matrix with sparse SiNWs (see Figure 9a) was filled with an active material (see Figure 9b,c): PEDOT + MnO_2_ were electrodeposited on the SiNWs in an electrolyte containing EDOT, Na_2_SO_4_, sodium dodecyl sulfate (SDS), and MnSO_4_ at a constant voltage of 2 V in a two-electrode cell. The current collector layer was prepared as follows:

- Reduced graphene oxide (rGO), synthesized by hydrazine reduction, was deposited by ELD from an rGO solution for 10 min (see Figure 9d);
- Subsequently, the PEDOT-PSS layer incorporated with Ag nanowires (AgNWs:PEDOT-PSS = 1:2) was spin-coated (see Figure 9e,f).

The rGO not only improves the capacitance of the electrode by providing EDL capacitance but also greatly promotes the transfer of electrons and ions. XRD analysis revealed that the birnessite-type MnO_2_ NPs had poor crystallinity. Nevertheless, the composite electrode material with low crystallinity exhibited higher ionic conductivity and shorter diffusion paths. Both of these properties are beneficial for improving capacitance, reaching 101 mF/cm^2^ with 81% retention after 2000 cycles.

In further works, Ni particles were electrodeposited on SiNWs in a two-electrode system at a constant current of 3 mA, from an aqueous electrolyte containing NiSO_4_, NH_4_Cl, and SDS. The Ni particles connected with each other to generate a highly conductive layer that prevented silicon from oxidation/corrosion, largely improving the stability of SiNWs and the electrode.

**Figure 10:**
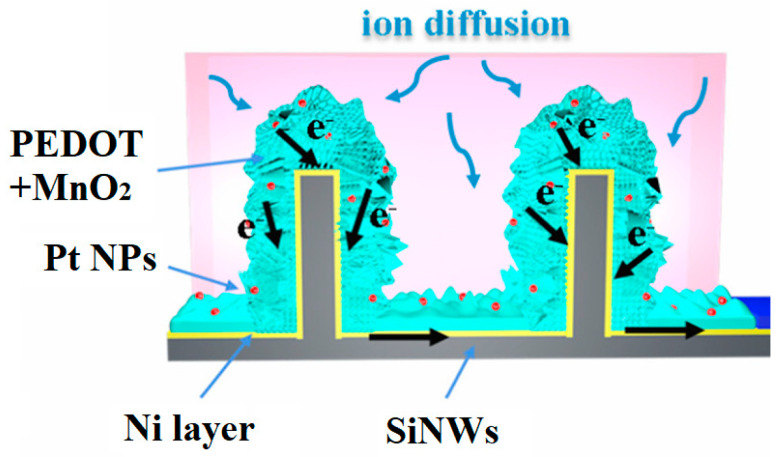
Schematic illustration of the SiNWs/Ni/PEDOT + MnO_2_/Pt electrode. Ref. [74] is reproduced with permission, Copyright © 2022, American Chemical Society.

In work [74], a PEDOT + MnO_2_ layer was deposited on SiNWs/Ni structure by the one-step co-electrodeposition method as ascribed in [53]. Finally, Pt NPs decorated the electrode by immersing it in the ethanol solution containing H_2_PtCl_6_ for 1 min, followed by annealing at 180 °C for 30 min in vacuum. After decoration with Pt NPs, the conductivity of the composite was improved, and the surface became more hydrophilic, favoring ion/charge transport. EIS measurements also confirmed that Pt NPs decreased ion diffusion resistance and improved electron transfer. This electrode design (see Figure 10) provided a capacitance of 207 mF/cm^2^, losing 5% of its value after 2000 cycles.

In [59], the capacitance value was increased to 328.6 mF/cm^2^ (with 79% retention after 7000 cycles) after MnO_2_ electrodeposition on SiNWs/Ni under a constant current of 0.003 A in a two-electrode system, with a platinum sheet utilized as the counter electrode. The electrolyte contained 0.4 M MnSO_4_, 0.025 M SDS, and 0.1 M Na_2_SO_4_. Excessive mass-loading of MnO_x_ not only hindered electron/ion transfer due to increased distance but also diminished contact areas between the active material and electrolyte, consequently impeding ion/electron transfer within the electrode. Notably, some cracks were observed within the dense MnO_x_ layer. These cracks in the MnO_x_ layer provided more active sites and facilitated electrolyte penetration, thereby enhancing the electrode’s capacitance.

PEDOT was electropolymerized on SiNWs/Ni, followed by dipping in KMnO_4_ solution [60]. Platinum particles were introduced between PEDOT and MnO_x_, which not only enhanced the mass loading of MnO_x_ but also improved the conductivity of the composite. After spin-coating a PEDOT-PSS layer doped with silver nanowires on the composite, a hierarchical core–shell electrode comprising active materials and a current collector was formed. As a result, the capacitance of 352 mF/cm^2^ was achieved with 85% retention after 2000 cycles.

Ppy and nickel–cobalt sulfides were co-electrodeposited on nickel-particle-passivated SiNW arrays to achieve a gravimetric capacitance of 1602 F/g [94].

SiNWs/Ni/NiCoSe-rGO composite provided a gravimetric capacitance of 2268 F/g, which is equivalent to an areal capacitance of 1973 mF/cm^2^ [95]. The ECD of the nickel–cobalt selenide nanosheets and rGO was carried out at voltages ranging from 1.6 to 0 V with a scan rate of 15 mV/s in an electrolyte solution containing nickel and cobalt nitrates, selenium dioxide, lithium chloride, and rGO. The prepared NiCoSe layer was composed of aggregated nanoparticles, generating a porous layer. The abundant pores within the active layer favored full contact with the electrolyte, facilitating ionic diffusion. The hierarchical core–shell structures not only afforded abundant electrochemical reaction sites but also provided short distances for electron/ion transport, largely improving the charge transfer kinetics during the charging/discharging process.

### 6.5. Comparison

All the data from the considered works (as well as from works in which the achieved capacitance is less than 1 mF/cm^2^) are summarized in Table 1 in order of increasing capacitance value.

Clearly, VLS is superior to MACE in terms of using the obtained structures as electrodes of microcapacitors. Interestingly, two studies deviate from this pattern: VLS-synthesized SiNWs, when coated with crystalline chromium nitride [28] or amorphous nickel boride [25], exhibit capacitances greater than 100 mF/cm^2^. Both materials are pseudocapacitive. CrN layers reduce charge transfer resistance (this is atypical for pseudo-capacitive materials). More attention should be given to such metal-containing coatings, as well as to the relationship between the capacitive properties of SiNW-based composites and the crystalline structure of coatings.

MnO_x_ is the most investigated transition metal oxide used as a coating for pSi/SiNWs electrodes. EPD-deposited MnO_2_ provides a slight increase in overall capacitance [33] compared to the precipitation method (ELD), which yields higher results [30,67]. A direct comparison of the two methods was also performed in the work [34], which confirmed the advantages of ELD. Moreover, the electroless process occurs at room temperature, making it compatible with microelectronic processing technology.

The other main types of coatings used are carbon-based, primarily graphene, and conductive polymers. Their advantage is high conductivity, which improves charge transfer. Researchers also note that a hierarchical core–shell structure provides protection from oxidative electrolyte contact and a shorter ion path, but a high degree of adhesion is necessary in such structures.

Table 2 summarizes the key coatings for pSi/SiNWs, as well as their main benefits and drawbacks regarding electrochemical performance.

Cyclic stability is an important characteristic that requires specific consideration. The Shen group’s concept, when sparse SiNWs act as a scaffold to be filled with active material, allows for superior capacitive performance. The authors attribute the cycle durability to the following:

- the smart design without consideration of the passivation/corrosion of the SiNWs in aqueous solution and the synergistic effects of the core–shell configuration and the combination of PsAg and rGO [53];
- cracks on MnO_x_ can effectively alleviate volume variations in MnO_x_ during electrochemical cycling [59];
- rGO underlayer used for NiCoSe coating [95].

However, noticeable losses are observed at average cycling times (less than 10,000 cycles) and need to be confirmed.

MnO_x_ loses capacitance as a result of consecutive reduction to soluble Mn^2+^ ion [104]. Very stable cycling performance has been achieved with MnO_2_ hierarchical sphere-based electrodes [73]. The results reveal that tiny nanostructures are generated and disappear during the cycling test, causing the reorganization of MnO_x_ and resulting in a transformation of its morphology. The loss of active materials in the form of nanostructures clearly causes capacitance degradation. Currently, it is still unclear what triggers and controls such morphological evolution.

Nevertheless, MnO_x_ possesses low cyclic stability. For instance, the MnO_x_/C/SiNW structure retains 84% of its initial capacitance after 5000 cycles [55]. However, C/SiNWs demonstrate significant capacitance degradation [49]. These losses probably arise not from MnO_x_ dissolution, but from SiNW instability. Indeed, as seen in Table 1, for SiNW capacitance values greater than 100 mF/cm^2^, cyclic measurements do not exceed 15,000 cycles; for values greater than 200 mF/cm^2^, they do not exceed 7000 cycles with about 20% capacitance loss.

Conversely, 100% capacitance retention (or even higher) is observed for pSi (DRIE [35] or anodic [64,66] etched) with carbon coatings. These materials have been little investigated in the literature, while studies on SiNWs clearly outnumber those on pSi. This is likely due to the rapid development of MACE, a simple and accessible method. Certainly, the progress achieved allows us to consider that pSi is a promising material for on-chip supercapacitor applications, especially for long-term stable performance, and needs to be further studied.

## 7. Devices

Micropower sources have been in development since the 1980s. In 2003, the fabrication of microcapacitors was proposed in a study [105], causing a wave of interest in the field. After the report of an ultrahigh-power, micrometer-sized supercapacitor based on onion-like carbon [106], graphene-based planar micro-supercapacitors advanced significantly [107]. Other carbon materials, such as CNTs and porous carbon [108], as well as transition metal oxides, conductive polymers, and MXenes, have also been successfully integrated into microdevices or chip-based systems for energy storage [109]. Recently, the last class of 2D coatings opened up an exciting new field of 2D inorganic functional materials due to their intrinsic electronic conductivity, superior hydrophilicity, rich surface chemistry, and layered structure [110].

The above review focuses on nanostructured silicon electrodes and summarizes data on surface capacitance (which is important for on-chip applications as opposed to gravimetric ones) and the factors influencing it. PSi/SiNW-based microelectrochemical capacitors offer scalability and integration of on-chip energy storage components, but as discussed below, fabrication methods for such devices are currently underdeveloped.

**Figure 11:**
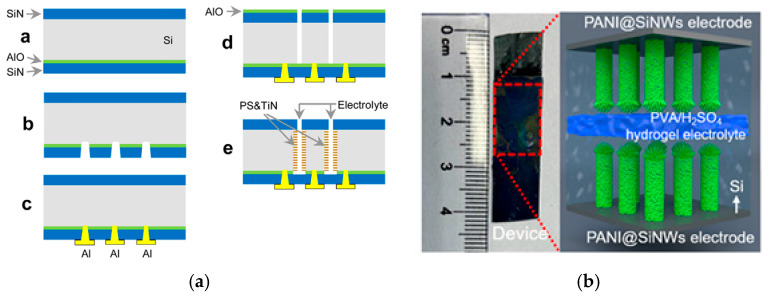
(**a**) Fabrication steps of the in-chip supercapacitor. (**b**) Schematic of the symmetric solid-state on-chip supercapacitors based on PANI/SiNW electrodes. (**a**) Ref. [78] is reproduced with permission, Copyright © 2016 The Authors. (**b**) Ref. [54] is reproduced with permission, Copyright © 2020, American Chemical Society.

Figure 11a [78] illustrates the fabrication of the in-chip supercapacitor device (a detailed description is provided in the Supplementary Materials, Section S3). To the best of our knowledge, the solid-state supercapacitor structure shown in Figure 11a has only been proposed in one study [78]. Most of the reported microcapacitors are fabricated by sandwiching the gel electrolyte between two pSi/SiNW-based electrodes, as shown in Figure 11b.

Table 3 contains data for some of the reported devices. The capacitance values for a single electrode (measured using a three-electrode electrochemical cell) are provided for comparison (certainly, these values are significantly higher than the device’s capacitance). As can be seen, energy and power densities can be adjusted by the properties of the electrodes and the measurement conditions.

Interestingly, the last two results are obtained in organic and aqueous electrolytes (works [55] and [95], respectively). Generally, organic electrolytes are preferable to aqueous ones because ionic liquids are stable over a much wider voltage window. This improves the theoretical maximum energy and power densities [49], which scale with the square of the potential window. For comparison, the authors of work [55] report a higher energy density (20–146 μWh/cm^2^) in an organic electrolyte with a 3.5 V voltage window than in an aqueous electrolyte with a 1.6 V voltage window (34–109 μWh/cm^2^ [95]), although the capacitance in the former work is almost six times lower than in the latter. Furthermore, hydrogen ions formed by water dissociation can diffuse through the silicon crystal lattice and passivate its dopants, thereby reducing the silicon matrix conductivity [111].

**Figure 12:**
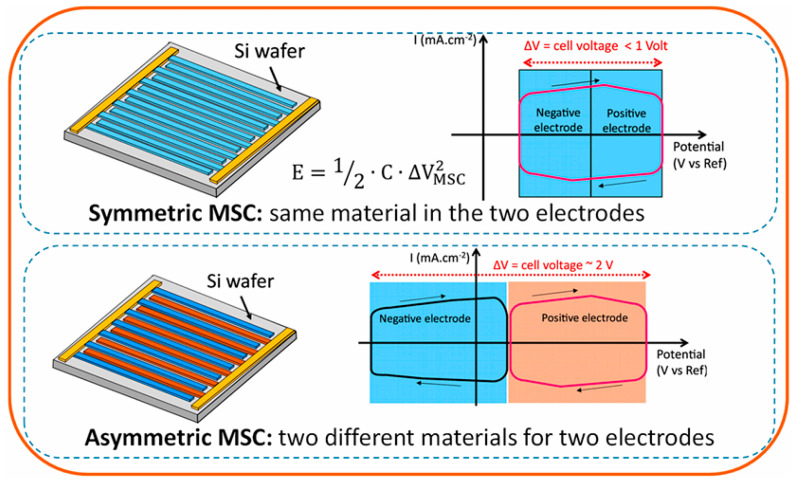
MSC based on interdigitated topologies with a symmetric configuration or an asymmetric configuration. Ref. [112] is reproduced with permission, Copyright © 2023, The Authors.

A sandwich-type electrode connection is not suitable for on-chip applications. Further development of on-chip microcapacitors also requires studying the features of devices with planar topology (e.g., as shown in Figure 12).

The electrochemical window can be extended when two different active materials are used in the negative and positive electrodes to create an asymmetric configuration (as shown in Figure 12), combining the potential windows of both electrodes [112]. Using conventional lithography, the gap between the electrodes should be reduced to micrometers, greatly increasing the resultant performance due to shorter diffusion paths [109]. Furthermore, it is essential to develop scalable and cost-effective preparation techniques for electrode materials to facilitate their large-scale production and commercialization [113]. Photo- or electron resists are unstable in HF solutions. Chemically resistant dielectric pattern layers must be applied to serve as both an etch mask and an insulator between the silicon substrate and the electrical contact. Although conventional microfabrication can reach resolutions of up to micrometers, high resolution can be hindered by multi-stage deposition processes. This requires a compromise between achieving the best electrochemical performance and using the simplest technological approaches to keep costs down when scaling up. To address compatibility challenges and accelerate the translation of research findings into practical applications, innovative approaches such as printable conductive inks should be prioritized [114].

The performance of the microcapacitors is influenced not only by topology, the width of the electrochemical window, and electrical contact formation, but also by electrolyte properties. Liquid electrolyte systems are poorly suited for on-chip integration or the satisfactory performance of embedded microelectronic components. In the reported pSi/SiNWs microcapacitor devices, gel polymers are mainly used, except in work [115], in which a solid polymeric separator is proposed. Since silicon-based anodes in advanced lithium-ion batteries have been studied and developed more extensively, we can apply their experience. General conclusions from reviews [116,117] that also apply to capacitors are as follows:

The drawback of all-solid-state electrolytes stems from inadequate interfacial contact between the solid electrolyte and the electrode surface. This leads to substantially elevated interfacial resistance and generally lower ionic conductivity.

Quasi-solid-state electrolytes employ gelation strategies to strike a balance between liquid- and solid-state characteristics. This achieves synergistic optimization of ionic conductivity and mechanical robustness. Additives such as ceramic and propylene carbonate and artificial interlayers have been shown to improve the structural integrity of the electrode/electrolyte interface.

## 8. Conclusions

On-chip electrodes for microcapacitors are a task that is of growing interest to researchers, as both alternative energy and portable devices are very relevant issues today. A summary of the literature data, including their compilation and analysis, leads us to conclusions about the most effective way to obtain the best electrochemical characteristics of pSi/SiNWs:

• Aligned MACE-etched SiNWs are better than twisted ones synthesized by VLS.
• Herringbone-like architecture of nanostructured silicon is preferred.
• Nanopores in pSi are more successful compared to micropores.
• The use of organic ionic liquids is preferable to aqueous electrolytes (quasi-solid-state electrolytes are promising).

The capacitance increases together with the growth of pSi depth/SiNW length, but the maximum depth/length can be limited by the following:

• The difficulty of completely impregnating the structure with the electrolyte;
• The difficulty of completely covering long SiNWs/deep pSi with additional coatings;
• The lengthened pathway for electron diffusion.

SiNWs provide the achievement of high capacitance values, while pSi structures are very promising for long-term stable performance. Therefore,

• The cyclic stability, especially for Shen’s and Maboudian’s group approaches, should be additionally investigated;
• More coatings (carbon, primarily graphene-based, conductive polymers, and metal compounds, especially oxides) should be tested to coat pSi;
• The influence of DRIE depth on pSi capacitance should be studied.

Most of the reported results are devoted to the materials of the electrodes. For further development of on-chip microcapacitors, it is necessary to study the features of performing devices. On the way to on-chip microcapacitors, many problems still need to be solved, both in their creation (micropatterning, insulation, and selection of a suitable electrolyte) and in their operation (cyclic stability and particle diffusion).

It is also necessary to use integral formulas to calculate the capacitance, as well as energy and power densities from GCD data, when the GCD curves deviate from the linear pattern.

## Figures and Tables

**Table 1:** Capacitance data, measurement conditions, and electrode materials extracted from various works.

Ref.	C, mF/cm^2^	Measured	Matrix (Method)	Coating (Method)	Electrolyte	Cap. Retention	
						%	Cycles
[36]	0.013	at 10 mV/s	SiNWs (VLS)	–	EMIM-TFSI *	98	1k
[96]	0.021	at 10 mV/s	11.9 μm SiNWs (MACE)	–	1 M TEABF_4_	83	0.5k
[97]	0.031	at 0.25 mA/cm^2^	20 μm SiNWs (VLS)	–	EMIM-TFSI	–	–
[32]	0.038	at 0.25 mA/cm^2^	5 μm SiNWs (VLS)	3 nm Al_2_O_3_ (ALD)	EMIM-TFSI	96	1000k
[50]	0.051	at 5 μA/cm^2^	50 μm SiNWs (VLS)	–	TEABF_4_ *	97	200k
[98]	0.108	at 5 mV/s	5 μm SiNWs (VLS)	diamond (CVD)	PMPyrr-TFSI	~93	10k
[99]	0.18	at 2.2 mA/cm^2^	SiNWs (VLS)	–	N_1114_TFSI	70	3000k
[47]	0.2	at 10 mV/s	pSi (AE)	–	0.25 M TEABF_4_	–	–
[100]	0.274	at 50 mV/s	2.7 μm SiNWs (MACE)	–	0.1 M Li_2_SO_4_	74	1k
[101]	0.3	–	1 μm SiNWs (VLS)	20 nm ZnO (ALD); 10 nm Al_2_O_3_(ALD)	–	–	–
[62]	0.32	–	43 μm pSi (AE)	Au	20% H_2_SO_4_	–	–
[102]	0.75	at 0.14 mA/cm^2^	SiNW powder (VLS)	–	0.5 M TBABF_4_	80	1000k
[63]	0.99	at 100 mV/s	pSi (AE)	nanodiamond (CVD)	0.1 M KCl	–	–
[31]	1.25	at 1 mA/cm^2^	50 μm SiNTrs (2-step VLS)	–	–	80	1000k
[87]	1.5	at 10 mA/cm^2^	50 μm SINWs (VLS)	diamond (CVD)	Et_3_NH-TFSI *	65	1000k
[79]	1.55	at 2 mV/s	20 μm SiNRs (DRIE)	30 nm TiN (ALD)	1 M Na_2_SO_4_	95.2	2k
[51]	1.7	at 50 mV/s	SINWs (MACE)	SiC (CVD)	1 M KCl	95	1k
[88]	2	at 0.01 mA/cm^2^	60 μm SINWs (MACE)	DLC (EPD)	0.5 M LiClO_4_	90	16k
[33]	2.1	at 0.04 mA/cm^2^	SINWs (VLS)	MnO_2_ (EPD)	1 M Na_2_SO_4_	90	5k
[77]	4.38 *	–	6 μm pSi (AE)	TiN (ALD)	TEABF_4_	stable	5.5 k
[61]	6.4	at 20 mV/s	50 μm SINWs (VLS)	Al_2_O_3_ (ALD); PEDOT-PSS (drop casting)	0.5 M Na_2_SO_4_	95	500 k
[66]	8.16	at 1000 mV/s	15 μm pSi (AE)	FLG (Ni-assist CVD)	0.5 M Na_2_SO_4_	130	10k
[58]	8.5	at 1 mA/cm^2^	50 μm SINWs (VLS)	diamond (CVD); PEDOT (ElP)	N_1114_TFSI	80	15k
[103]	9.64	at 1 mA/cm^2^	pSi (AE)	NiO (sol-gel)	1 M NaOH	97	5k
[30]	13	at 0.4 mA/cm^2^	50 μm SINWs (VLS)	MnO_2_ (ELD)	LiClO_4_-PMPyrrBTA	91	5k
[27]	14	at 1 mA/cm^2^	SiNTrs (VLS)	Ppy (ElP)	PYR_13_TFSI	70	10k
[29]	17	at 100 mV/s	10 μm SINWs (VLS)	PEDOT (ECD)	TBABF_4_	–	–
[22]	19	at 5 mV/s	6 μm SINWs (MAAE)	RuO_2_ (ALD)	1 M Na_2_SO_4_	92	10k
[67]	21.3	at 1000 mV/s	10 μm SINWs (MACE)	MnO_2_ (ELD)	1 M Na_2_SO_4_	–	–
[48]	25.6	at 0.1 mA/cm^2^	17 μm SINWs MACE	nanocarbon (glucose pyrolysis)	1 M Na_2_SO_4_	75	25k
[90]	30	at 0.5 A/g	5 μm pSi	GLC (DHN pyrolysis)	PVA-H_2_SO_4_	75	1k
[26]	36.25	at 1 mA/cm^2^	SiNWs (VLS)	Ru NPs (ELD)	1 M Na_2_SO_4_	80	25k
[34]	81.6	at 5 mV/s	~5 μm SiTNR (DRIE)	TiN (ALD); MnO_2_ (ELD)	1 M Na_2_SO_4_	95.7	5k
[56]	87	at 5 mV/s	80 μm pSi (AE)	GLC (CVD)	3 M H_2_SO_4_	100	15k
[54]	95.8	at 10 mV/s	5.5 μm SINWs (MACE)	PANI (OxP)	1 M H_2_SO_4_	71.8	2k
[53]	100.98	at 1.5 mA/cm^2^	5.6 μm SINWs (MACE+TMAH)	PEDOT + MnO_2_ (ECD); rGO (ELD); AgNWs + PEDOT-PSS (spin-coating)	1 M Na_2_SO_4_	81	2k
[52]	106.1	at 1 mA/cm^2^	17 μm SINWs (MACE)	PPy (OxP); PEDOT (PhP)	PYR_13_TFSI	80.2	5k
[75]	110	at 1 mA/cm^2^	10 μm SINWs (MACE)	N-carbon (PDOP pyrolysis); NiO (ELD)	6 M KOH	81	4k
[35]	123 192	at 1000 mV/s at 1 mV/s	~20 μm SiTNRs (DRIE)	FC-CNT (CVD)	H_2_SO_4_	102	5k
[91]	130	at 10 mV/s	10 μm SINWs (MACE)	GNWs (CVD); PANI (ElP)	PVA-H_2_SO_4_	80	2k
[64]	145	at 5 mV/s	80 μm pSi (AE)	N-GLC (CVD)	3M H_2_SO_4_	93	20k
[25]	165.7	at 0.1 mA/cm^2^	several μm SINWs (VLS)	NiB (ELD)	PVA-Na_2_SO_4_	93	10k
[28]	180 31.8	at 5 mV/s at 1.6 mA/cm^2^	SiNWs (VLS)	CrN (magnetron sputtering)	0.5 M Na_2_SO_4_	92	15k
[74]	207.43	at 1 mA/cm^2^	6 μm SiNWs (MACE+TMAH)	Ni + PEDOT + MnO_2_ (ECD co-deposition); Pt NPs (ELD)	1 M Na_2_SO_4_	95	5k
[49]	325	at 1 mA/cm^2^	120 μm SINWs (MACE)	nanocarbon (CVD)	EMIM-TSFI	83	5k
[59]	328.6	at 1 mA/cm^2^	10 μm SINWs (MACE+TMAH)	Ni (ECD); MnO_2_ (ECD)	1 M Na_2_SO_4_	79	7k
[60]	352	at 2 mA/cm^2^	several μm SINWs (MACE + TMAH)	Ni (ECD), PEDOT (ElP), Pt NPs (ELD); MnO_2_ (ELD) AgNWs+PEDOT-PSS (spin-coating)		85	2k
[55]	381	at 4 mA/cm^2^	80 μm SINWs (MACE)	nanocarbon (CVD); MnO_x_ (ELD)	0.1 M EMIM-TSFI	84	5k
[95]	1973	0.87 A/cm^2^	10 μm SiNWs (MACE + TMAH)	Ni (ECD); NiCoSe-rGO (ECD)	6 M KOH	80.5	2k

* 1-Ethyl-3-methylimidazolium bis(trifluoromethylsulfonyl)imide (EMIM-TFSI); tetraethylammonium tetrafluoroborate (TEABF_4_); triethylammonium bis(trifluoromethylsulfonyl)imide (Et_3_NH-TFSI).

**Table 2:** Key coatings for pSi/SiNW electrodes and their features in the context of microcapacitor applications.

Coating	Characteristic	Advantage	Disadvantage and/or Expectations
NiB, CrN	pseudocapacitive	high capacitance	the capacitance of crystalline NiB needs to be explored
MnO_2_	pseudocapacitive	ELD is compatible with microelectronic technology	moderate capacitance
MnOx with underlayer (TiN or carbon)	highly conductive underlayer	mentioned above + high or ultra-high capacitance	low cyclic stability
Carbon	improved charge transfer, good adhesion	high capacitance, high cyclic stability (for pSi)	CVD methods are poorly compatible with microelectronic technology
PEDOT-PSS	gelationous-like structure	ultra-high long-term cyclic	moderate capacitance
PANI with GNW underlayer	synergetic effect of both coatings	high capacitance	low cyclic stability
In combined coatings:			
Pt NPS, rGO	improved charge transfer	ultra-high capacitance	long-term cyclic tests need to be carried out
NiCoSe	porous structure facilitates ionic diffusion		
MnO_x_	cracks on the MnO_x_ facilitate electrolyte penetration		

**Table 3:** Device characteristics for two-electrode supercapacitors reported in some works.

Ref.	C_electrode_, mF/cm^2^	Device	C_device_, mF/cm^2^	E_device_, mWh/cm^2^	P_device_, mW/cm^2^
[53]	100.98 (at 1.5 mA/cm^2^)	symmetric	24.7 (at 1 mA/cm^2^)	0.0034	2.652
[52]	106.1 (at 1 mA/cm^2^)	symmetric	46.5 (at 0.5 mA/cm^2^)	0.0146	0.375
[35]	123 (at 1000 mV/s) 192 (at 1 mV/s)	symmetric	178 (at 5 mV/s)	0.0115 0.0096	2 34.7
[91]	130 (at 10 mV/s)	liquid state solid state	84.4 (at 100 mV/s)	0.0117 0.0108	0.42 0.782
[74]	207.43 (at 1 mA/cm^2^)	symmetric	64 (at 1 mA/cm^2^)	0.2503 1.5115	0.0103 0.006
[59]	328.6 (at 1 mA/cm^2^)	asymmetric	95 (at 1 mA/cm^2^)	0.021 0.00835	0.7998 7.9446
[55]	381 (at 4 mA/cm^2^)	symmetric	49 (at 2 mA/cm^2^)	0.146 * 0.0204 *	0.128 * 14.4 *
[95]	1973 (0.87 A/cm^2^)	asymmetric	273 * (at 2.06 mA/cm^2^)	0.109 * 0.034 *	1.6 * 16.4 *

* Calculated from the gravimetric data given in the corresponding papers (See details in the Supplementary Materials, Section S4).

## Supplementary Materials

The following supporting information can be downloaded at: https://www.mdpi.com/article/10.3390/nano15231826/s1, Section S1: Data table for Figures 1a and 1c; Section S2: The equivalent electrical circuit of the porous electrode; Section S3: Fabrication steps of the in-chip supercapacitor; Section S4: Areal calculations from reported gravimetric data.

## Abbreviations

The following abbreviations are used in this manuscript:

pSi	Porous silicon
SiNW	Silicon nanowire
AE	Anodic etching
DRIE	Deep reactive ion etching
MACE	Metal-assisted chemical etching
VLS	Vapor–liquid–solid
SiNR	Silicon nanorod
NP	Nanoparticles
MAAE	Metal-assisted anodic etching
M	mol/L
CVD	Chemical vapor deposition
SM	Supplementary materials
EDL	Electrical double layer
CVA	Cyclic voltammetry
GCD	Galvanostatic charge–discharge
EIS	Electrochemical impedance spectroscopy
PANI	Polyaniline
GLC	Graphene-like coating
SEM	Scanning electron microscopy
TMAH	Tetramethylammonium hydroxide
ELD	Electroless layer deposition
XPS	X-ray photoelectron spectroscopy
TEM	Transmission electron microscopy
XRD	X-ray diffraction
ALD	Atomic layer deposition
EPD	Electrophoretic deposition
SAED	Selected area electron diffraction
PMPyrrBTA	1-methyl-1-propylpyrrolidinium bis(trifluoromethylsulfonyl)imide
EDX	Energy-dispersive X-ray
ECD	Electrochemical deposition
SiTNR	Silicon taper nanorod
N-carbon	N-doped carbon
PDOP	Polydopamine
OxP	Oxidative polymerization
MW	Microwave
DLC	Diamond-like carbon
FC	Fullerene-like carbon
CNT	Carbon nanotube
FLG	Few-layer graphene
ElP	Electropolymerization
DHN	2,6-dihydroxynaphthalene
SPD	Sharp pressure drop
PPy	Polypyrrole
PYR_13_TFSI	N-propyl-N-methylpyrrolidinium bis(trifluoromethanesulfonyl)imide
PEDOT	Poly(3,4-ethylenedioxythiophene)
TBABF_4_	Tetrabutylammonium tetrafluoroborate
N_1114_TFSI	Butyltrimethylammonium bis(trifluoromethylsulfonyl)imide
PSS	Poly(styrenesulfonate)
PhP	Photo polymerization
GNW	Graphene nanowall
SDS	Sodium dodecyl sulfate
rGO	Reduced graphene oxide
EMIM-TFSI	1-Ethyl-3-methylimidazolium bis(trifluoromethylsulfonyl)imide
TEABF_4_	Tetraethylammonium tetrafluoroborate
Et_3_NH TFSI	Triethylammonium bis(trifluoromethylsulfonyl)imide
