# Supplementary material for: Porous Silicon and Silicon Nanowires for On-Chip Supercapacitor Electrodes: A Review
Source: Nanomaterials (Basel). 2025 Dec 2;15(23):1826. doi: 10.3390/nano15231826 (PMC12693459; doi:10.3390/nano15231826)
Supplement: Supplementary file 1 [file nanomaterials-15-01826-s001.zip › nanomaterials-3993212-supplementary.pdf]

Supplementary Materials

<u>Porous silicon and silicon nanowires for on-chip supercapacitor electrodes: a review</u>

Daria M. Sedlovets*

Institute of Microelectronics Technology and High-purity Materials, Russian Academy of Science, Chernogolovka, Moscow District, 6 Academician Ossipyan str., 142432 Russian Federation

Corresponding author: sedlovets@iptm.ru (Daria Sedlovets)

Section S1. Data table for Figures 1a and 1c

Year	Figure 1a		Ref. number	Figure 1c	
	pSi	SiNWs		VLS	MACE
1999	0.2		47		
2007	0.32		62		
2005	0.99		63		
2014	4.4		77		
2018	8.2		66		
2018	30		89		
2022	87		56		
2025	145		64		
2017		0.013	36	0.013	
2020		0.021	96		0.021
2014		0.031	97	0.031	
2017		0.038	32	0.038	
2013		0.051	50	0.051	
2015		0.108	98	0.108	
2017		0.18	99	0.18	
2024		0.274	100		0.274
2013		0.3	101	0.3	
2019		0.75	102	0.75	
2016		1.25	31		1.25
2016		1.5	87	1.5	
2016	1.55		79		
2012		1.7	51		1.7
2022		2	88		2
2019		2.1	33	2.1	
2018		6.4	61		6.4
2016	9.6		103	9.1	
2015		13	30	13	
2015		14	27	14	
2019		17	29	17	
2017		19	22		
2019		21.3	67		21.3
2016		25.6	48		25.6
2019		36.25	26	36.25	
2017	81.6		34		
2020		95.8	54		95.8
2019		106.1	52		106.1

2021		110	75		110
2017	123	192	35		
2020		130	91		130
2024		165.7	25	165.7	
2019		180	28	180	
2024		207.43	74		207.43
2024		328.6	59		328.6
2022		352	60		352
2014		325	49		325
2017		381	55		381

Section S2. the equivalent electrical circuit of the porous electrode

Figure from ref. 44 is reproduced with permission, Copyright © 2020 The Authors.

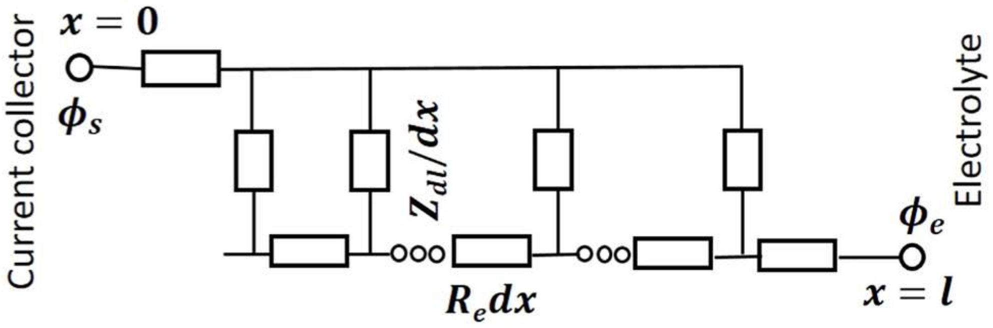

he upper line corresponds to the electronic phase, of which the electronic resistivity is zero. The bottom line corresponds to the electrolyte phase, of which the resistivity is Re.The total electrolyte resistance of the pore equal to Rel, where l is the length of pore. The specific impedance of the interface between electronic and electrolyte phase is denoted as Zdl. Between two nodes connected by an anionic resistance of Redx, the potential difference is given, according to the Ohm's law in frequency domain. At each node, the current change is equal to the current flowing from the electronic phase to the solution phase. At the other end of the pore, electrical current in the electrolyte phase cannot penetrate into the pore wall, namely, the electrical current is zero.

Section S3. Fabrication steps of the in-chip supercapacitor

Figure from ref. 78 is reproduced with permission, Copyright © 2016 The Authors.

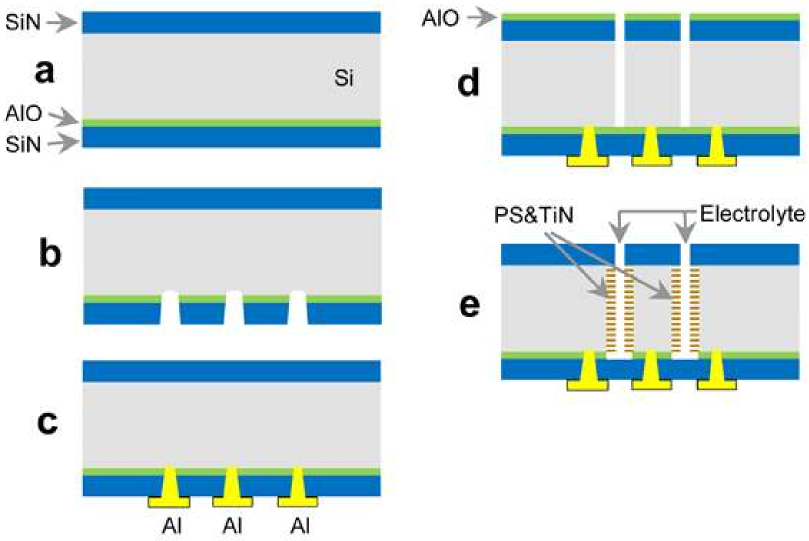

The process starts with ALD aluminium oxide layer (15 nm) and silicon nitride layer (1.5 mm thick) deposition which serves as an etch stop layer and supporting structure for the electrodes respecttively **(a)**. Contact openings are done through both layers **(b)** and supercapacitor electrode contacts are prepared by sputtering and patterning of aluminium **(c)**. Al_2O_3 is prepared by ALD on the top side of the wafer and patterned to act as a mask for anisotropic plasma etching through the wafer to form trench reservoirs for the electrolyte **(d)**. The next steps are pSi formation on the trench sidewalls and TiN coating by ALD **(e)**. Due to the conformal growth, provided by ALD, the TiN layer creates a short circuit at the bottom of the trench. The short circuit is removed by ion-beam etching resulting in supercapacitor device where two galvanically separated supercapacitor electrodes with electrolyte reservoir are inside silicon chip. The electrolyte reservoir is filled with an aqueous electrolyte (1 M NaCl).

Section S4. Areal calculations from reported gravimetric data

In references 55 and 95 from main text were not reported the area-normalized energy and power density data. However, the mentioned papers contained all the information necessary for the calculation.

Citing ref. 55, "the mass of MnOx/C/PSiNWs is calculated as 0.42±0.04 … based on the electrode area of 0.7 cm^2". The area-normalized energy and power density values can be obtained from the gravimetric ones as follows:

$$244\ \mathrm{Wh/kg} = 244 \times (0.42 \times 10^{-6}\ \mathrm{kg}) \times \left(\frac{1}{0.7\ \mathrm{cm}^2}\right) = 146.4\ \mu\mathrm{Wh/cm}^2$$

$$34\ \mathrm{Wh/kg} = 34 \times (0.42 \times 10^{-6}\ \mathrm{kg}) \times \left(\frac{1}{0.7\ \mathrm{cm}^2}\right) = 20.4\ \mu\mathrm{Wh/cm}^2$$

$$213\ \mathrm{W/kg} = 213 \times (0.42 \times 10^{-6}\ \mathrm{kg}) \times \left(\frac{1}{0.7\ \mathrm{cm}^2}\right) = 127.8\ \mu\mathrm{W/cm}^2$$

$$24\ \mathrm{kW/kg} = 24 \times 10^3 \times (0.42 \times 10^{-6}\ \mathrm{kg}) \times \left(\frac{1}{0.7\ \mathrm{cm}^2}\right) = 14.4\ \mathrm{mW/cm}^2$$

In Shen's work (ref. 95) the total mass loading was given as ~2.06 mg/cm^2. The area-normalized energy and power density values can be obtained by simply multiplying by the mass densities:

$$53.23\frac{\mathrm{Wh}}{\mathrm{kg}} = 53.23 \times 10^{-6} \times \left(2.06\frac{\mathrm{mg}}{\mathrm{cm}^2}\right) = 109\ \mu\mathrm{Wh/cm}^2$$

$$16.62\ \mathrm{Wh/kg} = 16.62 \times 10^{-6} \times (2.06\ \mathrm{mg/cm}^2) = 34\ \mu\mathrm{Wh/cm}^2$$

$$800\ \mathrm{W/kg} = 800 \times 10^{-6} \times \left(2.06\frac{\mathrm{mg}}{\mathrm{cm}^2}\right) = 1.6\ \mathrm{mW/cm}^2$$

$$8000\ \mathrm{W/g} = 8000 \times 10^{-3} \times \left(2.06\frac{\mathrm{mg}}{\mathrm{cm}^2}\right) = 16.4\ \mathrm{mW/cm}^2$$
